# Increased ROS Scavenging and Antioxidant Efficiency of Chlorogenic Acid Compound Delivered via a Chitosan Nanoparticulate System for Efficient In Vitro Visualization and Accumulation in Human Renal Adenocarcinoma Cells

**DOI:** 10.3390/ijms20194667

**Published:** 2019-09-20

**Authors:** Revathi Kavi Rajan, Mohd Zobir Hussein, Sharida Fakurazi, Khatijah Yusoff, Mas Jaffri Masarudin

**Affiliations:** 1 Department of Cell and Molecular Biology, Faculty of Biotechnology and Biomolecular Sciences, University Putra Malaysia, 43400 Serdang, Selangor, Malaysia; 2 Cancer Research Laboratory Institute of Biosciences, University Putra Malaysia, 43400 Serdang, Selangor, Malaysia; 3 Materials Synthesis and Characterization Laboratory, Institute of Advanced Technology, University Putra Malaysia, 43400 Serdang, Selangor, Malaysia; 4 Department of Human Anatomy, Faculty of Medicine and Health Sciences, University Putra Malaysia, 43400 Serdang, Selangor, Malaysia; 5 Department of Microbiology, Faculty of Biotechnology and Biomolecular Sciences, University Putra Malaysia, 43400 Serdang, Selangor, Malaysia

**Keywords:** chitosan nanoparticles, chlorogenic acid, chemopreventive, nanobiotechnology, drug encapsulated chitosan nanoparticles, in vitro accumulation of encapsulated chlorogenic acid, 1,1-Diphenyl-2-picrylhydrazine assay, MTT assay

## Abstract

Naturally existing Chlorogenic acid (CGA) is an antioxidant-rich compound reported to act a chemopreventive agent by scavenging free radicals and suppressing cancer-causing mechanisms. Conversely, the compound’s poor thermal and pH (neutral and basic) stability, poor solubility, and low cellular permeability have been a huge hindrance for it to exhibit its efficacy as a nutraceutical compound. Supposedly, encapsulation of CGA in chitosan nanoparticles (CNP), nano-sized colloidal delivery vector, could possibly assist in enhancing its antioxidant properties, in vitro cellular accumulation, and increase chemopreventive efficacy at a lower concentration. Hence, in this study, a stable, monodispersed, non-toxic CNP synthesized via ionic gelation method at an optimum parameter (600 µL of 0.5 mg/mL of chitosan and 200 µL of 0.7 mg/mL of tripolyphosphate), denoted as CNP°, was used to encapsulate CGA. Sequence of physicochemical analyses and morphological studies were performed to discern the successful formation of the CNP°-CGA hybrid. Antioxidant property (studied via DPPH (1,1-diphenyl-2-picrylhydrazyl) assay), in vitro antiproliferative activity of CNP°-CGA, and in vitro accumulation of fluorescently labeled (FITC) CNP°-CGA in cancer cells were evaluated. Findings revealed that successful formation of CNP°-CGA hybrid was reveled through an increase in particle size 134.44 ± 18.29 nm (polydispersity index (PDI) 0.29 ± 0.03) as compared to empty CNP°, 80.89 ± 5.16 nm (PDI 0.26 ± 0.01) with a maximal of 12.04 μM CGA loaded per unit weight of CNP° using 20 µM of CGA. This result correlated with Fourier-Transform Infrared (FTIR) spectroscopic analysis, transmission Electron Microscopy (TEM) and field emission scanning (FESEM) electron microscopy, and ImageJ evaluation. The scavenging activity of CNP°-CGA (IC_50_ 5.2 ± 0.10 µM) were conserved and slightly higher than CNP° (IC_50_ 6.4±0.78 µM). An enhanced cellular accumulation of fluorescently labeled CNP°-CGA in the human renal cancer cells (786-O) as early as 30 min and increased time-dependently were observed through fluorescent microscopic visualization and flow cytometric assessment. A significant concentration-dependent antiproliferation activity of encapsulated CGA was achieved at IC_50_ of 16.20 µM as compared to CGA itself (unable to determine from the cell proliferative assay), implying that the competent delivery vector, chitosan nanoparticle, is able to enhance the intracellular accumulation, antiproliferative activity, and antioxidant properties of CGA at lower concentration as compared to CGA alone.

## 1. Introduction

The history of therapeutic compounds discovery from various sources (i.e., natural, semi-synthetic, and synthetic) can be traced back to the 1800s. One such compound include the naturally produced 5-O-caffeoylquinic acid (5-CQA) compound or commonly known as chlorogenic acid (CGA); an ester of caffeic acid and L-quinic acid [1,2]. This phytochemical is one of the active compounds utilized in Chinese traditional herbal medication plant, *Lonicera japonica* Thunb. [3]. The compound can be lavishly extracted from coffee beans, tea, vegetables (potato, artichoke, tomatoes, eggplants), fruits (berries, apples, prunes, kiwi), and herbaceous plants (*Artemisia capillaris*, *Ilex paraguariensis*, *Pimpinella anisum*, *Cymbopogon citratus*) [4,5,6,7,8].

CGA is an extensively studied plant secondary metabolite with potent antioxidant strength and other conceivable therapeutic properties. The presence of a catechol structure serves as the primary binding site for free radicals [9,10]. CGA is able to chelate metal ions, inhibit lipid oxidation, and scavenge free radicals, oxygen and nitrogen species (superoxides, hydroxyl, peroxy, peroxynitrate and hypochlorous acid) [2,10,11,12,13]. Lu et al. (2000) observed that CGA had enhanced scavenging effects compared to Vitamin C and E; with two to three-fold DPPH radicals scavenging effects and 10 to 30-folds superoxide anion radical scavenging effects [14].

Therewithal, studies have also shown potential participation of CGA as a nutraceutical compound in both in vitro and in vivo applications; which includes prevention against microorganisms [15,16,17], and potentially lowered the risk of certain health conditions such as obesity [18], cancer [19], hypertension [20], diabetic [21], myocardial infarction [22], and so on. However, regardless of ongoing studies on therapeutic properties of CGA, there are other impediment factors decelerating its pharmacological effects.

The compound integrity of CGA is influenced by temperature and pH. CGA was reported to undergo isomerization or possibly other chemical reactions (esterification, hydrolysis, etc.) under the exposure of high temperature (time-dependent), and at basic and neutral pH [23,24,25,26,27]. This, in return, affects the synthesis process and its nutraceutical trait. This polyphenol was also reported to exhibit poor cellular permeability [2,28,29,30]. Olthof et al. (2001) and Gonthier et al. (2003) perceived that only about 33% of ingested CGA was efficiently absorbed into the small intestines; the remainder are lost along the gastrointestinal tract or decomposed by microflora in the cecum [28,29]. Likewise, Farah et al. (2008) also reported similar findings; only 30% of CGA was absorbed into blood circulation; inferring the bioavailability and efficacy of CGA are affected in this circumstance, in spite of its therapeutic potential [1]. Besides, CGA also appeared to show non-toxic attribute in in vitro condition [31].

Hence to confront these limitations, administration of a high dosage was used [1] but in which led to observable events of severe side effects such as DNA damage [32], cardiovascular complications [33], inflammatory and oxidative stress injuries [34], disruption in natural oxidant and antioxidant balance mechanisms [34], as well as vomiting, asthma, pruritus, anxiety, diarrhea, liver damage and kidney injury [34,35]. Additionally, the lack of knowledge of CGA therapeutic mechanisms also halts research that ventures further into its pharmacological functions [36].

Alternatively, nanoparticles incorporated drug delivery systems (nano-DDS) have been proposed as an auxiliary avenue to deliver CGA to the targeted sites in controlled released mechanism with conserved antioxidant and therapeutic strength, minimized administration dosage, and lowered side effects [8,31,37,38,39]. This study employs one of the most commonly adapted nanomaterials in nano-DDS, chitosan nanoparticles (CNP); synthesized via ionic gelation between protonated amine groups, -NH_3_^+^ of chitosan (CS) and anionic phosphate groups, -PO_4_^3−^ of cross-linker tripolyphosphate (TPP) [40,41]. Due to its biocompatible, biodegradable, and simple and mild synthesis process, CNP has been widely opted for in pharmaceutical industries as a safe vector to deliver therapeutic drug compounds, genes, proteins, peptides, DNA, RNA, vaccines, antigens, and different types of bio-payloads [42,43,44].

Collectively, this vector is able to protect the cargo from degradation by phagocytosis and circumvent drug resistance mechanisms, deliver therapeutic drugs to the targeted sites, provide an alternative route for insoluble drugs to permeate cells, improve the bioavailability and therapeutic efficacy of drugs in the living system, upturn the enhanced permeability and retention (EPR) effect, participate in sustained released rates of the drugs, and conserve the pharmacodynamics effects and in vivo stability of drugs [40,45,46,47,48,49]. Vila et al. (2004)—concerning the nasal administration of tetanus toxoid encapsulated CNP in mice model—showed a prominent high stimulation and enduring humoral immune response compared to tetanus toxoid vaccine alone [50].

This study was outlined based on the objective of enhancing the in vitro accumulation and cytotoxicity effect on human kidney cancer cell line (786-O), and improvise the antioxidant activity of chlorogenic acid by encapsulating into fabricated stable, monodispersed, colloidal CNP as a delivery vector. The physicochemical characterizations, antioxidant strength, and in vitro cytotoxicity activities of CGA and CNP encapsulated CGA hybrid were analyzed and optimized for better intracellular efficacy of CGA. Additionally, the in vitro accumulation of CGA was studied using fluorescently labeled CNP (*f*CNP). Hence, validating the encapsulated CGA has high potential as a drug delivery vector in pharmaceutical applications.

## 2. Results

### 2.1. Characterization of Synthesized CNP and Determination of Optimal Parameters

**Figure 1:**
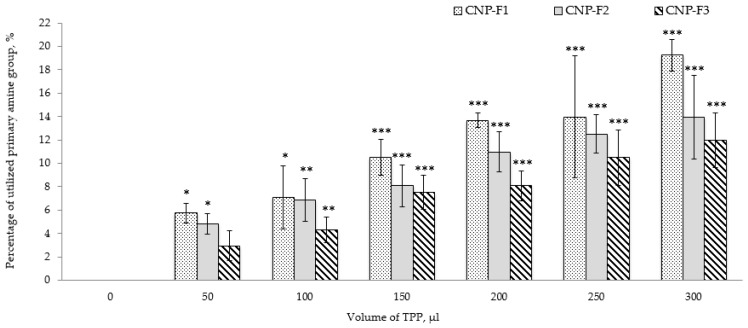
Influence of TPP volume on percentage of utilized amine group in combined graph bar CNP-F1, CNP-F2, and CNP-F3. The percentage of utilized primary amine (-NH_3_^+^) groups increases with TPP addition. The figure suggests that with increasing TPP volume, more phosphate ions, -PO_4_^3−^ groups were available to readily interact with -NH_3_^+^ groups to form CNP, hence reducing the availability of free -NH_3_^+^ groups. Error bars represent the standard deviation (SD) of three independent replicates of the experiment. * shows degree of significant difference, *p* < 0.0001 compared to control, 0 µL of TPP volume.

**Figure 2:**
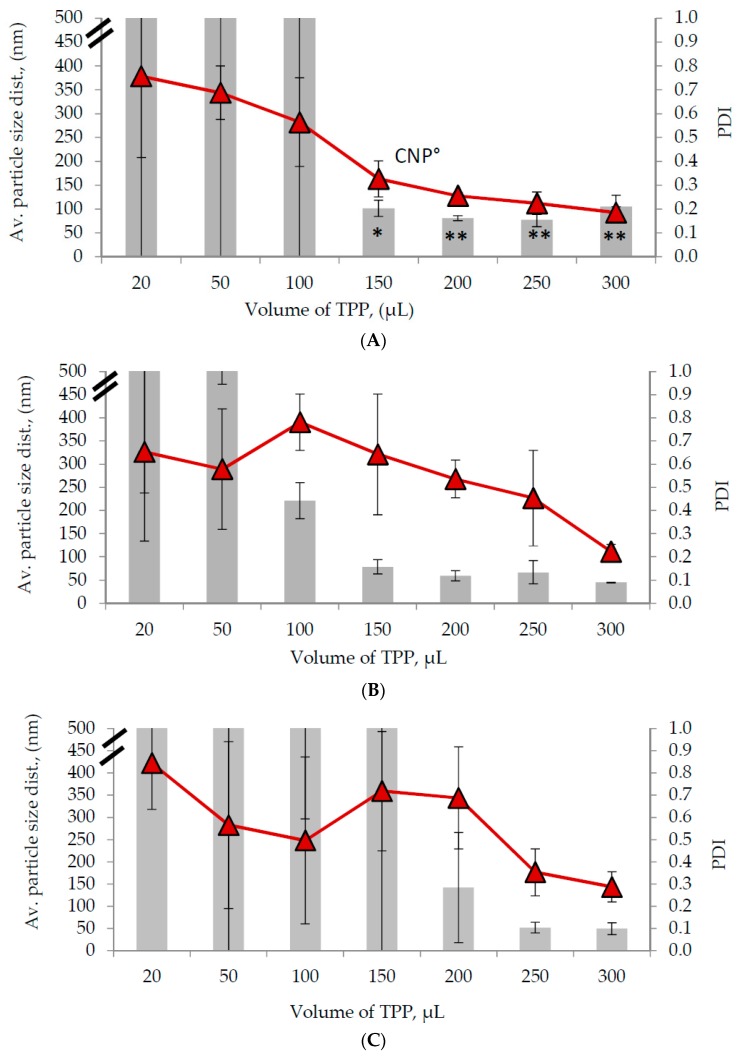
The influence of TPP volume of (**A**) CNP-F1, (**B**) CNP-F2, and (**C**) CNP-F3 on average particle size distribution (diameter in nm, nm), and polydispersity index (PDI). The average particle size distribution was illustrated in graph bar and PDI was illustrated in line graph. CNP° represents the optimal parameter required to synthesize stable and evenly distributed nanoparticle sizes. Error bars represent the SD of three independent replicates of the experiment. * shows degree of significant difference, *p* < 0.0015 compared to 20 µL of TPP volume.

CNP were formulated using three parameters as described in Table 1. The influence of TPP volume on the percentage of utilized primary amine groups (-NH_3_^+^), particle size distributions, and the stability of the nanoparticles depicted in Figure 1 and Figure 2. Following addition of a small volume of 1–3% of acetic acid, the primary amine groups (-NH_2_) of CS were protonated [51] spontaneously cross-linking with multivalent anionic phosphate groups (-PO_4_^3−^) of TPP to form hydrophilic CNP. However, not all -NH_3_^+^ groups participated in the cross-linking reaction. Shown in Figure 1, TNBS assay detected unutilized -NH_3_^+^ groups and subsequently confirms the formation of nanoparticles. On an overall basis, the percentage of utilized -NH_3_^+^ in formation of nanoparticles increased with the TPP volumes.

The percentage of utilized -NH_3_^+^ graph showed an increase of 13.50% utilized -NH_3_^+^ (from 5.75 ± 0.82% to 19.25 ± 1.36%) as the TPP volume increased from 50 µL to 300 µL in CNP-F1. Likewise, the utilized -NH_3_^+^ percentage in CNP-F2 and CNP-F3 increased 9.12% (from 4.83 ± 0.89% to 13.95 ± 3.60%) and 9.01% (from 2.96 ± 1.29% to 11.97 ± 2.34%), respectively, suggesting that at lower volumes of TPP, lesser -PO_4_^3−^ groups were available to cross-link with -NH_3_^+^; however, this circumstance overturned as the TPP volume increased. More -PO_4_^3−^ groups were present to interact with -NH_3_^+^ groups to form CNP, hence increasing the percentage of utilized -NH_3_^+^. A similar corresponding trend was also perceived previously by Masarudin et al. (2015) and this data correlated with particle size and dispersity of synthesized CNP as observed in Dynamic Light Scattering (DLS) analysis [52].

Based on Figure 1, a general decrease was observed in average CNP sizes (diameter in nm, nm) and PDI values as the volume of TPP increased for all three formulations. At lower TPP volumes, high variable particle size and PDI value data were observed across the three parameters due to insufficient availability of -PO_4_^3−^ to cross-link with -NH_3_^+^. However, the particle size and PDI value lessened as the TPP volume was increased as more -PO_4_^3−^ became available to perform internal cross-linking with -NH_3_^+^ within the CS particles, thus forming a more compact CNP and lower agglomeration rate. These results further supported the TNBS analysis.

Synthesis of nanoparticle sized between 50 nm to 100 nm, a range volume of 200 µL to 250 µL TPP for CNP-F1 (Figure 2A), 150 µL to 250 µL TPP for CNP-F2 (Figure 2B), and 250 µL of TPP for CNP-F3 (Figure 2C) were needed. CNP with particle size ranged between 50 and 100 nm were favored because at these size ranges, nanoparticles were able encapsulate more drugs, showed better cellular permeability across tight junctions and cell membranes, and gave extended in vivo circulation half-life [42,52,53,54,55]. PDI value represents the stability and dispersity of nanoparticle sizes; lower PDI (less than 0.3) which indicates stable and monodispersed nanoparticles was preferred compared to higher PDI (more than 0.7) which shows unstable and uneven distribution of particle sizes or agglomeration [56]. Based on Figure 2, the preferred PDI values was only observed in 200 µL TPP for CNP-F1 (Figure 2A), and at 300 µL TPP for CNP-F2 (Figure 2B) and CNP-F3 (Figure 2C).

**Figure 3:**
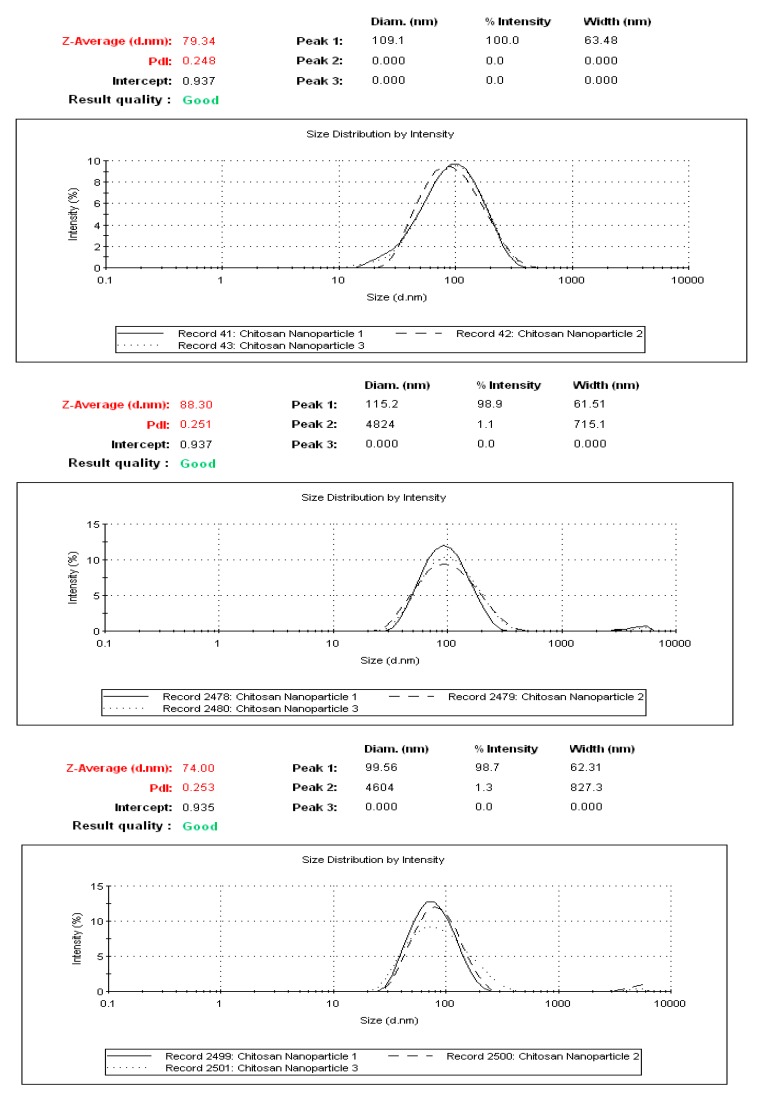
Reproducibility of DLS analyses for the optimal parameter, CNP° using 600 µL CS (0.5 mg/mL) and 200 µL TPP (0.7 mg/mL). Consistency in synthesizing nanoparticle with average 80.89 ± 5.16 nm with PDI value of 0.26 ± 0.01 were observed in all three replicates; showing that at this parameter, stable and monodispersed CNP° were consistently able to be synthesized.

Collectively, formation of stable and monodispersed CNP within the size range of 50–100 nm, was only be attained from two CS: TPP volume ratios of 3:1 and 2.4:1 of CNP-F1. CNP-F1 at 3:1 CS: TPP volume ratio parameter yield an average CNP particle size distribution of 80.89 ± 5.16 nm and a PDI value of 0.26 ± 0.01, while at 2.4:1 CS: TPP volume ratio produced a CNP of 77.36 ± 13.99 nm and 0.22 ± 0.05 PDI value. A consistency in small-sized, stable, monodispersed CNP with a low PDI value was perceived from using 200 µL TPP and 600 µL CS in CNP-F1(Figure 3) and was subsequently chosen as the optimum parameter to form CNP (denoted as CNP° in subsequent sections).

In this study, CS concentration below 1.5 mg/mL and TPP below 1.0 mg/mL was used as better CS solubility and CNP stability was attained [57]. Similarly, CS at pH 5 and TPP at pH 2 were opted as previously described by Masarudin et al. (2015) [52]. At pH 5 approximately 90% of deacetylated primary amine groups of CS is protonated, causing electrostatic repulsion between adjacent CS molecules [58,59,60] and the presence of inter-molecular hydrogen bonding between the CS molecules keeps the CS chain in an intact form [52,57,61].

In aqueous conditions, TPP dissociates into phosphate ions (PO_4_^3−^) and hydroxide ions (OH-) [62]. Studies reported that TPP at pH 2 enable the nanoparticles to participate controlled release mechanism compared to higher pH (i.e., 5 and 8.5) [63]. Besides, at lower pH (i.e., pH 2), only phosphate ions are formed, thus lesser -NH_3_^+^ are deprotonated by anionic TPP, which leads to the formation of smaller and monodispersed CNP [52,61,62]. Conversely, at higher pH, non-specific ionic gelation between both CS chains and existing CNP occur causing formation of agglomerated and larger-sized CNP due to presence of both PO_4_^3−^ and OH- [52,61,62]. The centrifugation step which was carried prior to addition of TPP to CS is important to discard larger and agglomerated CS molecules and TPP, and thus ease the formation of small and monodispersed nanoparticles [52].

### 2.2. Physicochemical Characterization of CNP°-CGA Nanoparticles

The nutraceutical and antioxidant potential of CGA is overshadowed by poor compound stability and poor intracellular permeability. Administration of high dosage to encounter these deficits lead to many unexpected side effects [28,35,64]. Therefore, a CNP-mediated drug delivery system was adapted as a vector to encapsulate CGA as a cargo to improve its intracellular therapeutic efficacy. The close proximity between CGA and -NH_3_^+^ of CS chains aid encapsulation of the compound within the core of CNP° via electrostatic interaction [65]. Successful formation of CNP°-CGA hybrids was achieved using CGA concentrations 2 µM, 10 µM, and 20 µM determined through particle size distribution and PDI analysis, CGA encapsulation efficacy (CGA-EE%), and CGA loading (CGA-L, µM) surmised in Table 2.

**Figure 4:**
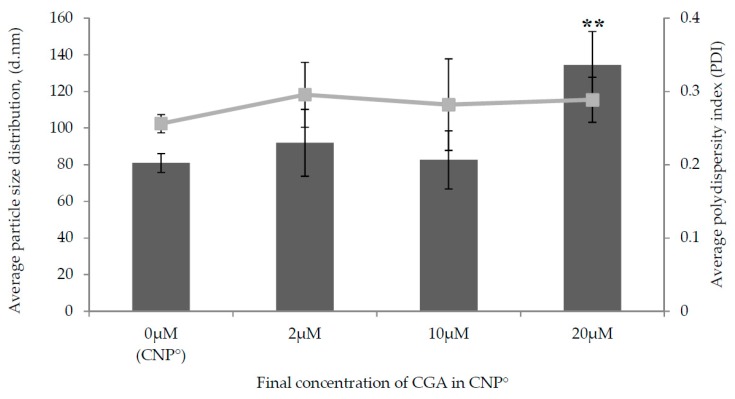
Effect of different final concentrations of CGA on average particle size distribution (nm) and polydispersity index, (PDI) of CNP°. The average particle size distribution is illustrated in bar graph and PDI is illustrated in line graph. Error bars represent the SD of three independent replicates of the experiment. * shows degree of significant difference, *p* < 0.0086 compared to control, CNP°.

A prominent expansion in particle size was observed in CNP°-CGA samples as shown in Figure 4 upon encapsulation of 20 µM of CGA (134.44 ± 18.29 nm). This was a 66.20% increase in size compared to 2 µM (91.99 ± 18.28 nm) and 10 µM (82.60 ± 15.81 nm) CGA with only 13.72% and 2.11% of increase, respectively; possibly due to a higher availability of CGA for encapsulation. It was suggested that the drug-nanoparticle hybrid between the size range of 100–200 nm has a better 4-fold increased cellular uptake in cancer cells, enhanced encapsulation due to small surface area to volume ratio, partake in slow drug release rate mechanism, and could escape from spleen and liver filtration as compared to particles sized less than 50 nm and more than 300 nm [55,66,67,68]. A small increase in PDI values were perceived upon encapsulation of 2 µM, 10 µM, and 20 µM of CGA; from 0.26 ± 0.01 (CNP°) to 0.30 ± 0.04 (an increase of 15.38%), 0.28 ± 0.06 (an increase of 7.69%), and 0.29 ± 0.03 (an increase of 11.54%), respectively. Presence of two types of particles in the hybrid, CNP° and CGA, was also inferred through this slight increase in PDI value.

Following the increase in CGA concentrations used for loading into CNP°; 2 µM, 10 µM, and 20 µM, CGA-EE% (percentage of CGA successfully loaded into nanoparticle) showed a declining trend from 74.43 ± 0.31%, 62.30 ± 0.05%, and 60.21 ± 0.03%, respectively, while CGA-L showed otherwise; 1.49 µM, 6.23 µM, and 12.04 µM, respectively (as can be seen in Table 2). CGA-L is much more significant than CGA-EE% as it represented the amount of CGA loaded per unit weight of CNP°, encapsulation of 20 µM CGA in CNP° was found to be better compared to other concentrations as higher amounts of CGA were found to be encapsulated into per unit CNP° weight. Evidently, formation of CNP°-CGA hybrids following loading of 200 µL of 100 µM (initial concentration) CGA, portrayed to be the optimal parameter as compared to 10 µM and 50 µM due to its significant particle size expansion, formation of stable, monodispersed CGA-nanoparticles hybrid, higher quantity of CGA encapsulated in per unit weight of CNP°.

According to Nagda et al. (2008) and Pavanveena et al. (2010), one factor that influences the amount of drug being entrapped within the nanoparticle is the drug to polymer ratio [69,70]. The higher this ratio, the lower the EE%. This inference ties well with this study, whereby three different ratios of CGA to CS were analyzed, i.e., 1:300 (for 2 µM), 1:60 (for 10 µM), and 1:30 (for 20 µM), showing that the EE% decreased as the CGA:CS ratio increases. Consequently, CGA loading increased along with concentration of the compound, indicating that more compound was able to be loaded per unit weight of CNP.

### 2.3. Morphological Characterization of CNP° and CNP°-CGA Samples

**Figure 5:**
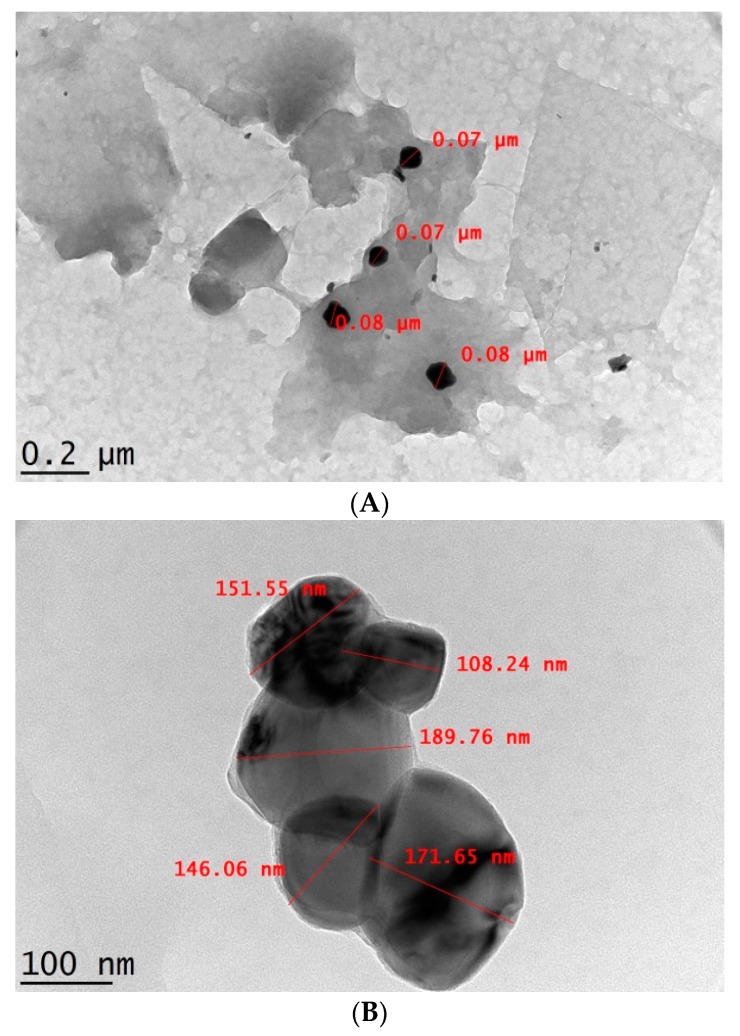
Images of Transmission Electron Microscopy (TEM) morphological analysis of (**A**) CNP° and (**B**) CNP°-CGA. Particles appeared as irregular spherical-shape for all parameters used.

Irregular, spherical-shaped and well dispersed CNP° and CNP°-CGA accompanied by particle size range of 70–80 nm and above 100 nm, respectively, were observed through Transmission Electron Microscopy (TEM) analysis in Figure 5A,B. The particles of CNP°-CGA were found to be stacked on one another with no apparent agglomerations of nanoparticles. Similar CNP morphology finding were reported previously by Raja et al. (2015) following loading of siRNA [71]. The CNP° appeared darker in Figure 5A due to scattering or absorption of electrons by compact and condensed nanoparticles formed through ionic gelation between CS and TPP [72]. In contrast, following loading of CGA, the size of CNP° increased due to interactions between CS with TPP and CGA via ionic gelation and electrostatic interactions. Formation of lighter or less dense region of CNP°-CGA (Figure 5B) may be due to transmittance of more electrons [72], while the dark shades inside the CNP° could possibly illustrate the successful encapsulation of CGA.

**Figure 6:**
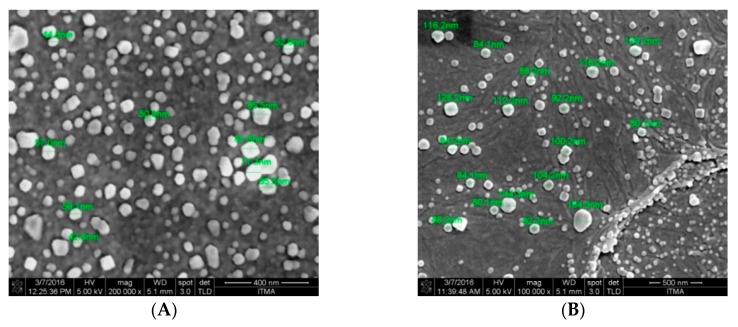
Images of FESEM morphological analysis of (**A**) CNP° and (**B**) CNP°-CGA. Particles appeared as irregular, dispersed spherical-shape for all parameters.

**Figure 7:**
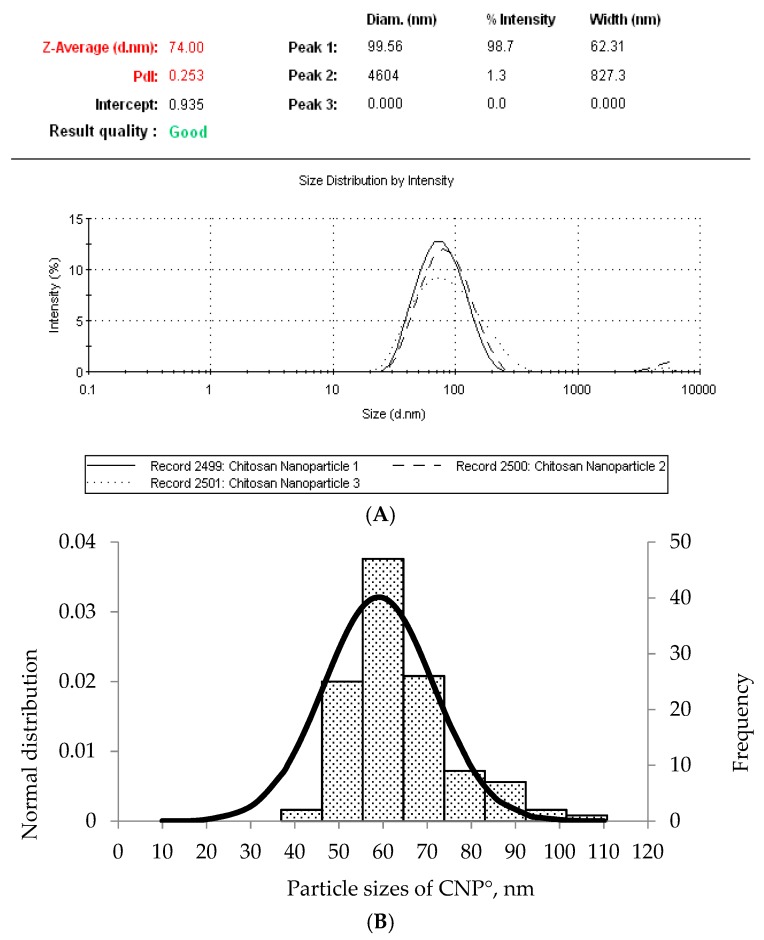
(**A**) Dynamic Light Scattering (DLS) result of CNP° and (**B**) normal distribution and frequency graphs of CNP° particle sizes (nm) obtained by measuring particle size in FESEM image in (Figure 6A) using ImageJ software. Comparable particle size range of CNP° was observed between DLS (80.89 ± 5.16 nm) and FESEM (50 nm and 70 nm). The slight difference between the two analysis is because DLS measures the particle size in hydrodynamic condition, while FESEM measures in a dry condition.

**Figure 8:**
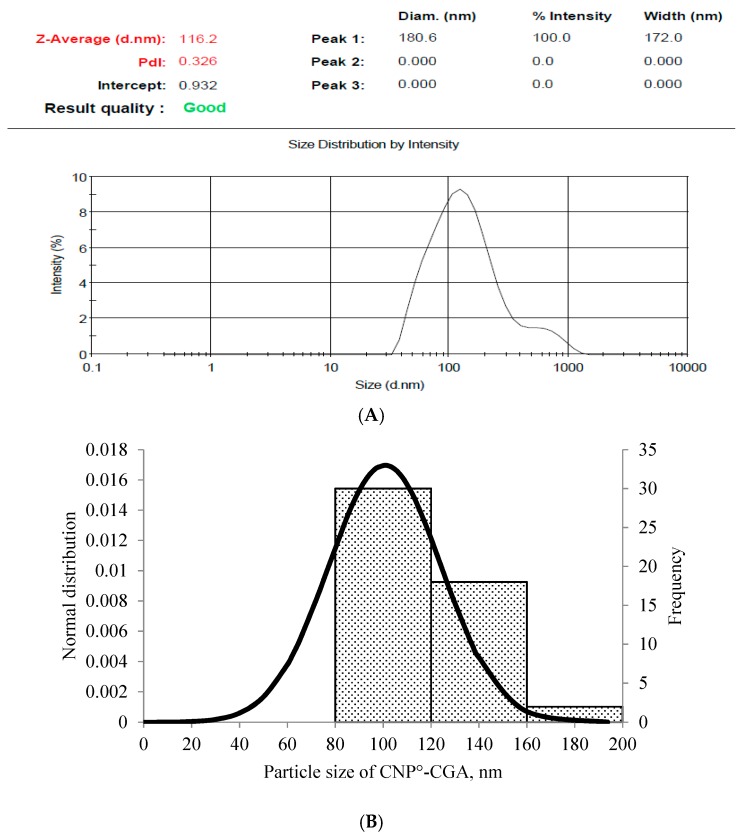
(**A**) DLS result of CNP°-CGA and (**B**) normal distribution and frequency graphs of CNP°-CGA particle sizes (nm) obtained by measuring particle size in FESEM image in (Figure 6B) using ImageJ software. Comparable particle size range of CNP° was observed between DLS (134.44 ± 18.29 nm) and FESEM (80 nm to 160 nm). The slight difference between the two analysis is because DLS measures the particle size in hydrodynamic condition, while FESEM measures in a dry condition.

The Field Emission Scanning Electron Microscopy (FESEM) images of CNP° and CNP°-CGA in Figure 6A,B showed irregular spherical-shaped nanoparticles with particle size ranging from 40–90 nm, and 80–160 nm, respectively. The morphological image of CNP° was found to be similar to studies by Luo et al. (2010), Chuah et al. (2013), and Ariff et al. (2017), hence verifying the formation of the nanoparticles [73,74,75]. Following encapsulation of CGA, the particle size of CNP° increased two-folds, complimenting to particle size increase from 80.89 ± 5.16 nm to 134.44 ± 18.29 nm in the DLS analysis, hence confirming the formation of CNP°-CGA hybrids. Consequently, particle distribution analysis was performed to further correlate FESEM and DLS results CNP° (Figure 7A,B) and CNP°-CGA (Figure 8A,B). The particle diameter of CNP° and CNP°-CGA in Figure 6A,B were manually measured and a normal distribution as well as frequency of the particle sizes were plotted, as shown in Figure 7B and Figure 8B. Based on the plotted graphs, the particle size range of CNP° and CNP°-CGA were 40–110 nm and 80–200 nm, respectively. While, the particle size frequency showed CNP° were mostly measured between 50 nm and 70 nm, and CNP°-CGA were in the range of 80 nm to 160 nm; double the size range of CNP°. These results correspond to the DLS results of CNP° (80.89 ± 5.16 nm) and CNP°-CGA (134.44 ± 18.29 nm).

The particle size distribution result of FESEM slightly differ from the DLS analysis (Figure 4 and Figure 6) as DLS measures the diameter of the nanoparticles based on scattering of light in its actual state (hydrodynamic condition) while FESEM is based on scattering of electrons in a dry condition [52]. Moreover, the consistency of particle size distributions and intact formation of nanoparticle shape were even perceived in both the FESEM images, implying that these properties were conserved upon encapsulation CGA.

### 2.4. Functional Group Analysis of CNP° and CNP°-CGA Samples

**Figure 9:**
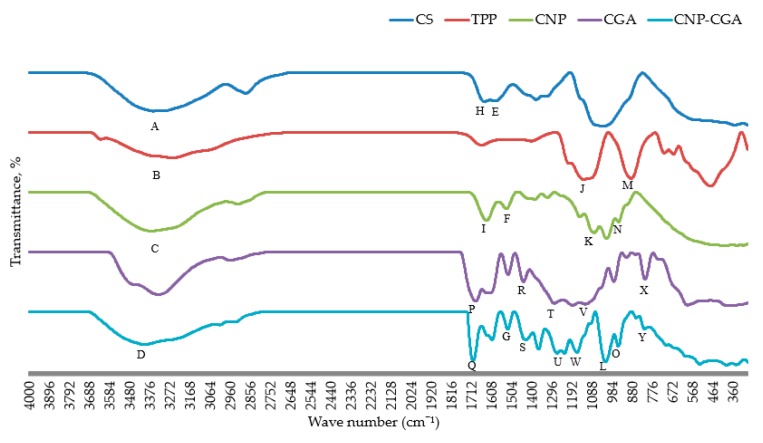
FTIR spectra of CS, TPP, CNP°, CGA, and CNP°-GCA. The successful formation of CNP° and CNP°-CGA were discerned through the presence of functional group peaks. Freeze dried samples were used to ensure sensitivity of analysis, also to remove non-specific background peaks. Alphabet labeling denotes important functional groups of samples, and is further elaborated in Table 3.

The functional groups attributed to FTIR spectrum of CS, TPP, CNP°, CGA, and CNP°-CGA were illustrated in Figure 9 and summarized in a tabulated form in Table 3. The -OH and -NH_2_ stretching vibration of CS at 3353.91 cm^−1^ was observed in CNP° at 3364.97 cm^−1^ and CNP°-CGA at 3398.61 cm^−1^ possibly due to presence of intermolecular hydrogen bonds during the formation of nanoparticles and encapsulated nanoparticles [60,76,77]. While in TPP, the -OH stretching vibration was perceived at 3253.92 cm^−1^.

Interestingly, two characteristic peaks of TPP; P=O stretching peak at 1126.89 cm^−1^ and asymmetrical stretching vibration of P-O-P peak at 883.70 cm^−1^ were also apparent in CNP° at 1073.91 cm^−1^ and 948.77 cm^−1^, respectively, and in CNP°-CGA at 1014.92 cm^−1^ and 949.14 cm^−1^ respectively [60,77,78,79]. These two peaks were not found in the CS spectrum; suggesting presence of these functional groups in CNP° and CNP°-CGA were influenced by the ionic gelation between phosphate moiety of TPP and primary amine groups of CS [8,76,77,80,81]. Besides, the CS peaks associated to C=O of amide I stretching (1641.06 cm^−1^) and N-H bending vibration in amide II (1587.00 cm^−1^) was identified in CNP° at peak 1630.94 cm^−1^ and peak 1527.82 cm^−1^, respectively, which was also perhaps due to TPP and CS cross-linking [60,76,77,78,82,83].

The encapsulation of CGA in CNP° vector was discerned via the presence of five peaks attributed to CGA in the CNP°-CGA spectrum, which initially were absent in the CNP° spectrum. Aromatic ring C=C stretching vibration at 1439.88 cm^−1^, C=O stretching vibration of carboxyl and ester group at 1687.62 cm^−1^, stretching vibration of C-O-C and C-O of carboxyl and ester group at 1279.45 cm^−1^ and 1185.17 cm^−1^, bending of C-H and COH at 1119.50 cm^−1^, and CH aromatic bending at 810.46 cm^−1^ of CGA spectrum were spotted in CNP°-CGA spectrum at 1428.52cm^−1^, 1700.27 cm^−1^, 1265.04 cm^−1^ and 1228.71 cm^−1^, 1163.43 cm^−1^, and 812.13 cm^−1^, respectively [31,38,39,84,85,86,87,88].

Additionally, an increase in transmittance percentages of the N-H bending of amide II peak in CS, 52.24%, and P-O-P asymmetrical stretching vibration peak in TPP, 22.73% was observed upon formation of CNP°, 72.08% and 49.86%, respectively; may indicate the ionic interaction between TPP and CS. However, scarcely any changes in transmittance percentage of those two functional groups were perceived in CNP°-CGA (70.60% and 41.68%, respectively), implying the energy transmittances were not affected by the electrostatic interactions between CGA and CS during the formation of CGA-nanoparticle hybrids. Hence, this supports the successful formations of CNP° and CNP°-CGA.

### 2.5. Assessment of Antioxidant Potentials of CGA and CNP°-CGA

**Figure 10:**
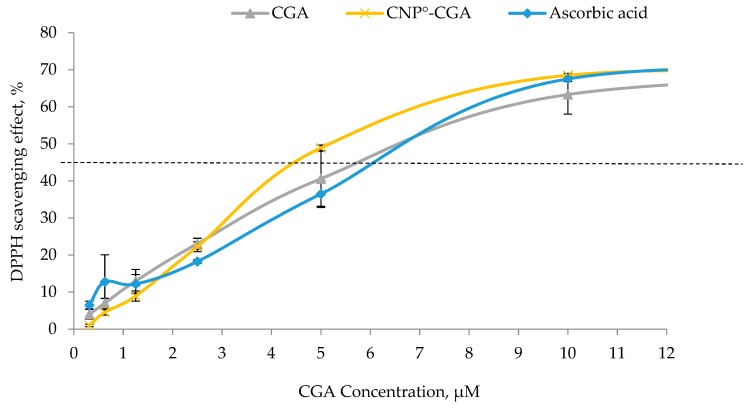
Antioxidant capacity of CGA, CNP°-GCA and ascorbic acid as standard at different concentrations. Error bars represent the SD of three independent replicates of the experiment. Dotted line indicates the IC_50_.

The ability of CGA and CNP°-CGA to reduce DPPH into DPPH-H form was measured and compared to access their respective antioxidant strength. The common standard reference, ascorbic acid, was used. The concentration for all three samples, CGA, CNP°-CGA and ascorbic acid were limited to 12 µM (on the graph) as the CGA-EE% as 60.21 ± 0.03%, assuming approximately 60% of CGA was successfully encapsulated. As shown in Figure 10, a dose-dependent DPPH scavenging trend was observed among CGA, CNP°-CGA, and ascorbic acid postulating more DPPH radicals was reduced along with an increase in antioxidants concentration [89,90,91].

Ascorbic acid reached its scavenging effect plateau point at 11 µM, with 70% of DPPH scavenged. Remarkably, similar scavenging plateau point was observed at CNP°-CGA; suggesting a maximum of 11 µM of CNP°-CGA was required to scavenge 0.1 mM DPPH—as can be observed from Figure 10—whilst the plateau point of CGA was not seen; instead the scavenging activity continued rising even after 12 µM. We postulate that CNP°-CGA has better antioxidant strength than CGA, as it could scavenge DPPH much faster with lower concentration.

CGA compound could actively partake in scavenging activity due to its two hydroxyl groups at *ortho* position of its catechol; as claimed by Valgimigli et al. (2008) [92]. However, the antioxidant strength of CGA was prominently slower than CNP°-CGA possibly due to its formation π-stacking between of catechol of CGA through strong OH-H or weak OH-π bonds; which might affect the transfer of H atom to scavenge the DPPH [93,94]. This phenomenon did not occur or minimized in CNP°-CGA as CNP° gives a good spatial orientation for CGA, perhaps the reason for better scavenging activity. Conversely, the scavenging activity of CNP° was below 10%; implying the vector itself is weak antioxidant as similarly reported [95,96] and therefore did not contribute towards the increased antioxidant efficiency in CNP°-CGA sample.

The IC_50_ values of pure CGA and CNP°-CGA, the minimum antioxidant concentration needed to scavenge 50% of DPPH radicals, are shown in Figure 10. Ascorbic acid displayed an approximate IC_50_ value of 6.5 µM similar to CGA_,_ while CNP°-CGA displayed a slightly lower IC_50_ of 5 µM. We deduced that the encapsulated CGA has a better antioxidant strength as its scavenging activity was still conserved, and comparatively less CGA concentration was needed to scavenge 50% of DPPH than CGA to achieve similar scavenging activity.

### 2.6. In Vitro fCNP°-CGA Uptake in Human Renal Adenocarcinoma 786-O Cells

Time-dependent in vitro uptake of FITC labeled CNP°-CGA (*f*CNP°-CGA) in 786-O cell line was studied through fluorescent microscopic and flow cytometry analyses. FITC has the ability to conjugate with the primary amine group of CS through covalent bonding without dissociating and provides a sensitive study on cellular accumulation efficiency of fluorescently labeled nanoparticles under the influence of time (30 min, 6 h, 24 h, and 48 h) [52,97]. Supplementary 1 showed that the uptake of *f*CNP°-CGA in 786-O cells was time-dependent, as the fluorescent intensity increased consequently with incubation time. Cellular uptake of *f*CNP°-CGA was detected as early as 30 min (Supplementary 1C) post-treatment with weak fluorescent signals outlining the cells membrane observed; a small portion of *f*CNP°-CGA being taken up by 786-O cells. The fluorescent intensity in the cells thereafter increased in 6 h (Supplementary 1D), 24 h (Supplementary 1E), and 48 h (Supplementary 1F) post-incubation treatments.

Conversely, smearing of the green fluorescent within the cells was observed at 24 h, and 48 h post-treatment, possibly due to the dissociation of FITC from CNP°-CGA [52]. It also indicated that CNP was effectively internalized by the cells; a slow degradation of CNP surface over time led to a release of encapsulated biomolecules (FITC and CGA) via a burst release model (releasing CGA close to the surface of CNP°) and erosion release model (releasing the encapsulated CGA) [98,99,100,101,102]. In in vitro studies, cultured cells propagated in media are exposed to high levels of lactic acid, which may decrease the intracellular pH environments. This lower pH condition protonates more amine groups of CNP° causing its encapsulated contents to be dissociated and released intracellularly [103,104,105]. Hypothetically, in in vivo condition the degradation of the vector likely be aided by the lysozyme activity or swell under the influence of endosome or lysosome pH [106,107,108,109]. On the other hand, the uptake of *f*CNP°-CGA into the 786-O cells was solely dependent on the nanoparticle and not influenced by presence of the FITC, as no fluorescent signal was detected in control cells or at 48 h of post-treatment with FITC only. This also meant that the fluorophore can only enter the cells by conjugation with the nanoparticle. Similar observations were observed in studies by Ma et al. (2003), Jia et al. (2009), and Masarudin et al. (2015) [27,52,110].

**Figure 11:**
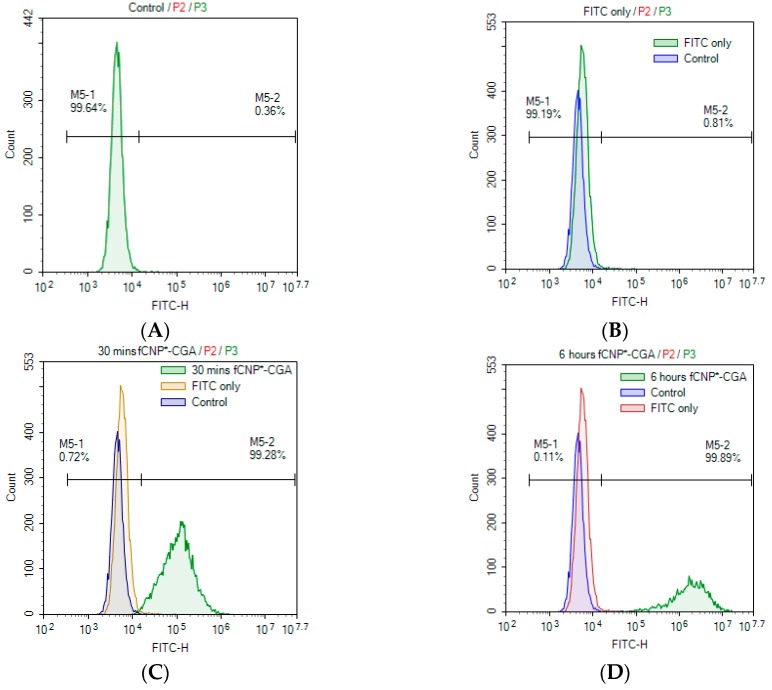

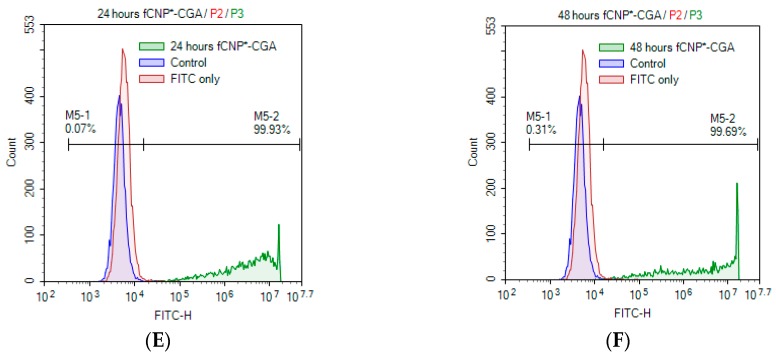
Flow cytometry study of in vitro accumulation of *f*CNP°-CGA in 786-O cells at (**C**) 30 min, (**D**) 6 h, (**E**) 24 h, and (**F**) 48 h. Overlap peaks of particular time point on (**A**) control and (**B**) FITC only peaks were shown in each figure to show the accumulation of *f*CNP°-CGA in 786-O cells. The accumulation of *f*CNP°-CGA in 786-O cells showed a time-dependent trend, indicated through the increase of fluorescent intensity over time. Accumulation of *f*CNP°-CGA was observed as early as 30 min (**C**) and subsequently increased 6 h, 24 h, and 24 h post treatment (**D**–**F**).

Complimenting fluorescent microscopic analysis, flow cytometry analysis was performed as shown in Figure 11. A shift in the peak was observed at 30 min post-treatment (Figure 11C), describing about 99.28% of cells were outlined or accumulated by *f*CNP°-CGA. The peak continued to shift and percentage increased as the incubation period was prolonged to 6 h (99.89%) (Figure 11D), 24 h (99.93%) (Figure 11E), and 48 h (99.69%) (Figure 11F); signifying persistency in uptake of *f*CNP°-CGA across 786-O cell membranes. A slight decrease in FITC intensity percentage at 48 h could probably be due to instrument error. Additionally, high-sharp peak at 24 h and 48 h post-treatments were possibly due to the limit of the instrument (FITC intensity of 10^7^). Hypnotizing that the uptake of *f*CNP°-CGA could prolong till cells reaches the saturation point without the instrument limit. The 48 h post-treatment of FITC only peak overlapped with the control peak (cells only) as shown in Figure 11B, deducing no fluorescent signal was detected in or around the 786-O cells and FITC did not influence the cellular uptake of nanoparticles.

One of the main deficits of CGA is its poor cellular permeability, halting this antioxidant-rich compound from exhibiting it chemopreventive mechanism. The hypothesis whereby nanoparticles could possibly aid this compound to improve its cellular permeability was proven through these two analyses, the microscopic viewing and flow cytometry analysis. These two studies are very crucial to demonstrate the facilitation of CNP° in internalizing CGA into cells. There is the possibility that as more CGA compounds are internalized within the cells, it could carry out its chemopreventive activity more efficiently without being expelled from the system as described by Olthof et al. (2001), Gonthier et al. (2003), and Farah et al. (2008) in their respective studies [1,28,29].

Furthermore, multiple studies have supported the idea that the uptake of drug-nanoparticles hybrids were mainly influenced by particle size and the cellular uptake route. In this study, CNP°-CGA were synthesized with an average size of 134.44 ± 18.29 nm which falls within the favorable size range (50–200 nm) for an efficient cellular uptake [66,68] most likely to be taken up through the cell membranes through clathrin-mediated endocytosis mechanisms [27,111,112,113].

### 2.7. Cytotoxicity Analysis of CNP°, CGA, and CNP°-CGA

**Figure 12:**
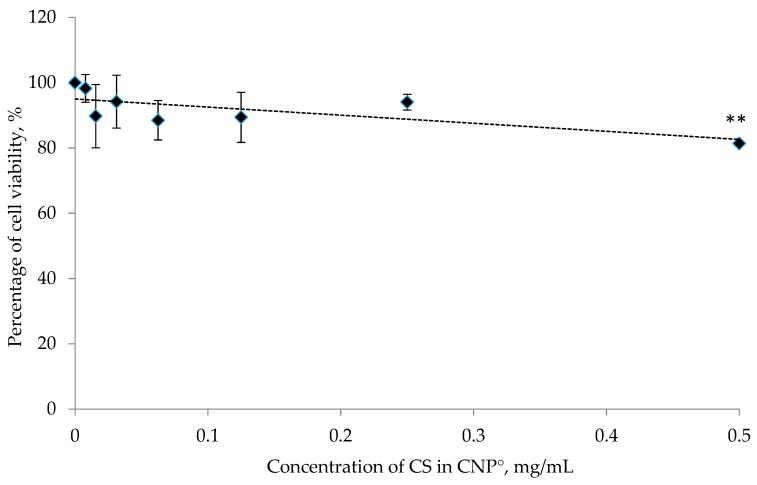
In vitro cytotoxicity test of CNP° at different concentrations. Error bars represent the SD of three independent replicates of the experiment. At the highest dosage, CNP° appeared to be non-toxic to 786-O cells upon 24 h post-treatment. * shows degree of significant difference, *p* < 0.0305 compared to control.

The MTT mediated cytotoxic assay was performed to study and compare the in vitro toxicity properties of the synthesized nanoparticles and drug-nanoparticles. Based on Figure 12, approximately 81.37 ± 0.84% of 786-O cells was viable 24 h post-treatment using CNP° and thus; exhibited non-toxic properties in cells at the highest dose. Viability continued to increase up to 100% as the concentration of CNP° decreased, a trend similarly reported by Jia et al. (2009) and Masarudin et al. (2015), thus suggesting CNP° as a potential safe drug delivery vector [52,110].

The naturally existing antioxidant-rich polyphenolic, CGA [2,114] has been actively studied as a chemopreventive agent. CGA has an ability to scavenge oncogenesis-inducing free radicals, facilitated by the catechol structure of CGA acting as the primary binding site for free radicals [9,10]. CGA has two to three-fold DPPH (1,1-diphenyl-2-picrylhydrazine) radicals scavenging activity and 10 to 30-fold superoxide anion radical scavenging activity as compared to vitamin C and E [14]. The binding sites of CGA does not only scavenges the oxygen and nitrogen species, but it also chelates metal ions, inhibits lipid oxidation, and hinders formation of free radicals [10,11,12,13,115].

Evidently, studies have reveal the ability of CGA to inhibit NF-κB transcription factor, repress DNA methylation, induce Nrf2 mediated antioxidant and detoxification enzymes (such as glutathione S-transferases (GST) and NAD(P)H: quinone oxidoreductase 1 (NQO1)) through antioxidant response element (ARE) to reduce oxidative stress cellular damages, and so on Reference [116,117,118]. Noratto et al. (2009) showed that CGA able to suppress the proliferation of MDA-MB-435, breast cancer cells, while Jin et al. (2005) managed to inhibit matrix metalloproteinase responsible for metastasis and invasion of cancer cell using CGA [119,120].

**Figure 13:**
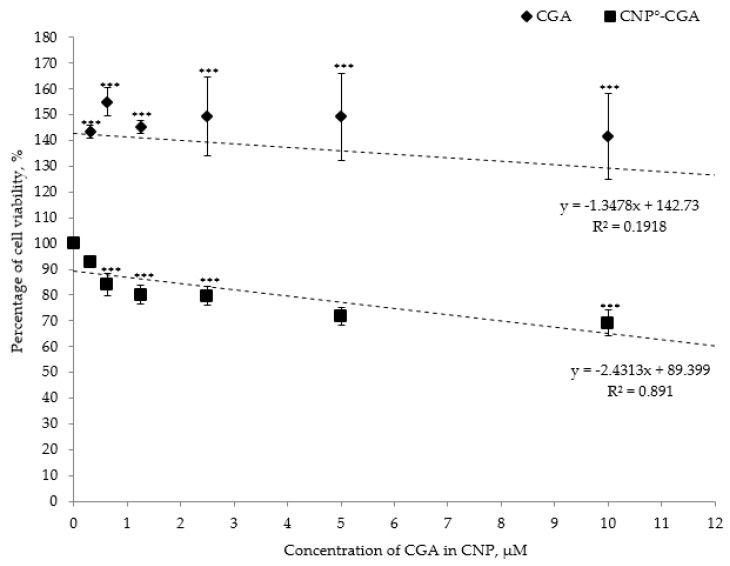
In vitro cytotoxicity test of CGA and CNP°-CGA at different concentrations. Error bars represent the SD of three independent replicates of the experiment. CGA alone was found to be non-toxic even at its highest dosage of 12 µM (24 h post-treatment). Conversely, a noticeable dose-dependent decrease in 786-O cell viability were observed upon encapsulation of CGA in CNP°. * shows degree of significant difference, *P* < 0.0001 compared to control.

Contrariwise, CGA is also known for its poor cellular permeability profile, loses its reliability as a potential nutraceutical compound [1,28,29]. Corresponding result was observed through the MTT assay analysis as shown in Figure 13; CGA appeared to be non-toxic to 786-O cell line. The percentages of cell viability were more than 100% at different CGA concentration ranged from 0 µM to 12 µM (Figure 13). Similar results were shown by Barahuie et al. (2013) and Barahuie et al. (2014), CGA acted as non-toxic compound towards 3T3 (mouse fibroblast normal cells), HeLa (human cervix adenocarcinoma) and MCF-7 (human mammary gland adenocarcinoma) cell lines even at maximum concentration of 50 µg/mL CGA [31,88]. We have strongly concluded that CGA is a non-toxic compound maybe due to its negative surface charges that hinders its uptake across 786-O cell membranes.

On the other hand, a significant shift in cytotoxicity effect on 786-O cell lines was observed following encapsulation of CGA in CNP° vector. The concentrations of CGA and CNP°-CGA used were again limited to 12 µM because of the maximum CGA-EE% of 60%. On the whole, the cell viability decreased in dose-dependent modus upon 24 h post-treatment of CNP°-CGA (Figure 13). Approximately 60.22% viable cells were detected at 12 µM of CNP°-CGA; suggesting that encapsulation of CGA able to provide a better route to deliver CGA intracellularlly and subsequently enhance its bioavailability and therapeutic efficacy.

The shift in cytotoxic effect of CGA following encapsulation in nanoparticles was possibly due to positive surface charge of the vector contributed by the unconjugated protonated amine groups, and this cationic charge was hypothesized to be conserved even after encapsulating CGA. Additionally, this feature of the vector enables it to participate clathrin-mediated endocytosis route to permeate across the anionic cell membrane [8,52,111,121,122]. Additionally, IC_50_ of CGA and CNP°-CGA were approximately calculated; 68.80 µM and 16.20 µM, respectively. An approximately 4-fold more CGA concentration was needed to achieve similar cytotoxic effect as CNP°-CGA. Deducing the encapsulated CGA potentially reduced the need for high dosage administrations of CGA to the cell line.

## 3. Materials and Methods

### 3.1. Materials

All materials were purchased and used without further purification, unless otherwise stated. Low molecular weight chitosan (CS) powder, sodium tripolyphosphate (TPP) powder, sodium dodecyl sulfate (SDS) powder, 2,4,6-trinitrobenzene sulfonic acid (TNBS) powder, L-ascorbic acid powder, 1,1-Diphenyl-2-picrylhydrazine 97%, (DPPH) powder, fluorescein 5(6)-isothiocyanate (FITC) powder, thiazolyl blue tetrazolium bromide (MTT) powder, phosphate buffered saline (10x) (PBS) solution, dimethyl sulfoxide (DMSO) solution, ethylenediaminetetraacetic acid (EDTA), and sodium carbonate powder, were purchased from Sigma-Aldrich (St Louis, MO, USA). Hydrochloric acid solution, sodium hydroxide pellet, and glacial acetic acid solution, and chlorogenic acid (CGA) powder were purchased from Friendmann Schmidt Chemicals (Germany). Methanol solution, reagent grade AR was purchased from QReC (New Zealand). RPMI medium 1640 (1×) solution, Fetal Bovine Serum (FBS) solution, antibiotic-antimycotic (100×) solution, and 0.25% Trypsin-EDTA (1×) solution were purchased from Gibco, Life Technologies (Grand Island, NY, USA).

### 3.2. Synthesis of Chitosan Nanoparticles, CNP

Chitosan nanoparticles (CNP) were prepared based on modified ionic gelation method of Calvo et al. (1997) [123]. One mg/mL stock solutions of chitosan (CS) and sodium tripolyphosphate (TPP) were prepared before diluting them to desired working solution as described in Table 1. CS stock solution was prepared by overnight stirring of 25 mg of low molecular weight chitosan powder in 1 mL of 1% acetic acid aqueous solution and 24 mL of sterile distilled water (sdH_2_O), while TPP stock solution was prepared simply by dissolving 25 mg of TPP powder in sdH_2_O. The working solutions of CS and TPP were diluted to three different concentrations as shown Table 1. Subsequently, pH of the CS, and TPP solutions were adjusted to pH 5 (using 1 M NaOH), and pH 2 (using 1 M HCl) respectively, prior to centrifugation at 4000 rpm for 45 min at room temperature. CNP of different formulations were synthesized by adding increasing volume of TPP cross-linkers ranging from 20 µL to 300 µL, to 600 µL of CS. The CNP samples were incubated at room temperature, and used in subsequent analysis.

### 3.3. Quantification of Free Amines in CNP Formulations

CNP was formed via ionic gelation between protonated primary amine groups (-NH_3_^+^) of CS and anionic phosphate groups (-PO_4_^3−^) of TPP cross-linker. A slightly modified TNBS (2,4,6-trinitrobenzene sulfonic acid) assay protocol of Masarudin et al. (2015) was adapted to quantify the percentage of unconjugated primary amine group (-NH_3_^+^) in CNP formulations [52]. Freshly prepared 0.1 M sodium carbonate (NaCO_3_) at pH 8.5, 10% sodium dodecyl sulfate (SDS), 1 M hydrochloric acid (HCl), and 0.05% TNBS in 0.1 M NaCO_3_ at pH 8.5 were used for this assay. Serially diluted CS solution using NaCO_3_ (0.1 M, pH 8.5) were used as standards. CNP solutions were prepared based on the formulations in Table 1. Hundred microliters of TNBS at 0.05% concentration was mixed thoroughly into 100 µL of CS sample (control) and CNP samples, and incubated in water bath at 37 °C for 3 h. Upon incubation, 100 µL of each sample, 100 µL of 10% SDS, and 75 µL of 1 M hydrochloric acid, (HCl) were pipetted into wells of 96-well plate. Mixture of 50 µL of 0.1 M NaCO_3_ at pH 8.5, 100 µL 10% SDS, and 75 µL of 0.1 M HCl solution was set as blank. The samples were analyzed at A_335_ using microplate reader (Bio-Tek Instrument, Winooski, VT, USA). The percentage of free amine group was quantified using the following formula;

(1) $$Free\;amine\;group,\;\%\;=\frac{Absorbance\;of\;sample}{Absorbance\;of\;control}\times 100\%$$

### 3.4. Measurement of Particle Size Distribution, and Polydispersity (PDI) of Nanoparticle Samples

The formation of nanoparticles, particle size distributions and PDI value of the synthesized CNP samples were measured via dynamic light scattering (DLS) analysis using a Malvern Zetasizer Nano ZS instrument (Malvern Instrument, UK). The prepared CNP samples were centrifuged at 13,000 rpm for 20 min and diluted in sdH_2_O at 60:40 (sdH_2_O: CNP) ratio before analyzing them using DLS. The best CNP formulation (denoted hereon as CNP°) was used for the subsequent analysis.

### 3.5. Synthesis of Chitosan Nanoparticles-Encapsulated Chlorogenic Acid (CNP°-CGA) and Determination of Optimum Parameters for CNP°-CGA Formation Using DLS and Encapsulation Efficiency Analyses

Chlorogenic acid (CGA) of 10 µM, 50 µM, and 100 µM initial concentration were prepared by dissolving the commercially purchased CGA powder in sdH_2_O. The CGA was then mixed with the best CNP formulation; CNP° to form CNP encapsulated CGA (CNP°-CGA). The particle size and PDI value of CNP°-CGA were analyzed via DLS method. While, to determine encapsulation efficiency (EE%), the CNP°-CGA was prepared and centrifuged at 13,000 rpm, for 30 min. The free, unloaded CGA were collected and the absorbance was measured at A_325_ using spectrophotometer (Thermo Scientific Genesys 840-208100 UV/Vis Spectrophotometer). The percentage of encapsulation efficiency (EE, %) and CGA loading (CGA-L, µM) were calculated using the following formulas;

(2) $$EE,\;\%=\frac{Absorbance\;of\;total\;CGA-Absorbance\;of\;unloaded\;CGA}{Absorbance\;of\;total\;CGA}\times 100\%$$

CGA − L, µM = EE% × CGA concentration(3)

The best CNP°-CGA parameter was then subsequently used in the following analyses.

### 3.6. Morphological Assessment of CNP°, and CNP°-CGA via Electron Microscopy

To discern the formation hybrid nanoparticles, Field Emission Scanning Electron Microscopy (FESEM) and Transmission Electron Microscopy (TEM) were employed. The best parameter of CNP° and CNP°-CGA were prepared as described earlier. The samples were placed on a stub and dried in incubator at 37 °C for two to three days. The samples were then coated with gold and viewed under Ultra High Resolution FESEM (Nova NanoSEM 230, FEI). The CNP° and CNP°-CGA images were then processed using ImageJ software to assess the particle size distribution. The size of CNP° and CNP°-CGA particles were measured, and a histogram bar on diameter and normal distribution curve were plotted. While, for TEM analysis, both the samples were immobilized on copper grids and air dried before examining under TEM (Tecnai TF20× -Twin, FEI) at MIMOS Berhad.

### 3.7. Characterization of Functional Groups via Fourier-Transform-Infrared (FTIR) Spectroscopy

FITR analysis was performed to study the molecular components and chemical functional groups present in a sample, in this case, CS, TPP, CGA, CNP°, and CNP°-CGA. All samples were prepared fresh and were freeze dried before sending them for FTIR analysis at Faculty of Sciences, UPM. The obtained FTIR spectrum results were further analyzed.

### 3.8. Evaluation of Free Radical Scavenging Activities of CGA Alone and CNP°-CGA

The free radical scavenging activity of CGA, and CNP-CGA were evaluated using a modified DPPH radical scavenging assay protocol described previously by Blois, 1958 [124]. CNP°, CGA, CNP°-CGA, and ascorbic acid (standard) solutions were freshly prepared prior the analysis. Two-fold serial dilutions were then carried out for all the samples in distilled water. A hundred microliters of each sample dilution were mixed together with 100 µL of 0.1 mM DPPH in a 96-well plate. A hundred microliters of DPPH at 0.1 mM was set as control. The samples were incubated at ambient temperature for 30 min in dark. The absorbance of the samples was read at the maximum absorbance of A_517nm_ using a microplate reader (Bio-Tek Instrument, Winooski, VT, USA).

(4) $$DPPH\;scavenging\;effect,\;\%=\frac{Absorbance\;of\;control-Absorbance\;of\;sample}{Absorbance\;of\;control}\times 100\%$$

### 3.9. Propagation and Maintenance of Human Renal Adenocarcinoma Cells, 786–O Cells

Human renal adenocarcinoma cells, 786-O were obtained from ATCC (USA). The cell lines were grown and maintained in a complete growth media, CGM (90% of RPMI media; 10% of fetal bovine serum, FBS; and 1% of antibody). The adherent cells were incubated at 37 °C, and 5% of carbon dioxide (CO_2_), and upon 80% to 100% confluency, the cells were used for subsequent cell treatment analysis or sub-cultured.

### 3.10. MTT-Based Cytotoxicity Analyses of CGA, CNP°, and CNP°-CGA

MTT assay was used to measure the cytotoxicity of the CGA, CNP°, and CNP°-CGA on 786-O cells. The 786-O cells were seeded on a 96 well plate at a volume of 100 µL, and a density of 10 × 10^3^ cells/per well. The cells were incubated at 37 °C for overnight. The next day, the CGM from each well was discarded and replaced with 100 µL of fresh CGM. Freshly prepared CNP°, CGA, and CNP°-CGA samples were diluted (two-fold dilution) in CGM and a 100 µL of each diluted samples were mixed into each well and the plate was left to incubate at 37 °C, 5% CO_2_ for 24 h. The untreated well was used as control, and well with media only was used as blank. After 24 h, the samples were discarded, and replaced with 170 µL of fresh CGM, and 30 µL MTT (5 mg/mL in 1× PBS). The plate was covered with aluminium foil and incubated for 4 h at 37 °C. Subsequently, 150 µL of the solution from each well was discarded prior to addition of 100 µL of DMSO to solubilize the formazan crystals. The absorbance was immediately read at A_570nm_ using a microplate reader. The percentage of cell viability was calculated using the following formula;

(5) $$Cell\;viability,\;\%=\frac{Absorbance\;of\;control-Absorbance\;of\;sample}{Absorbance\;of\;control}\times 100\%$$

### 3.11. Fluorescent Labeling of Chitosan Nanoparticles-Encapsulated Chlorogenic Acid, fCNP°-CGA

The primary amine groups of CS were covalently conjugated to 0.25 mg/mL of fluorescein isothiocyanate (FITC), which was dissolved in DMSO. The solution was incubated for 15 min. Successively, CGA, and TPP were added into the solution. Formed *f*CNP°-CGA was used in the following analyses.

### 3.12. In Vitro Visualization and Quantification of fCNP°-CGA in 786–O Cancer Cells

Human renal adenocarcinoma cells, 786-O were seeded on 6 well plate at a density of 1 × 10^5^ cells/per well, and incubated for 24 h at 37 °C and 5% of CO_2_. The following day, the CGM was removed; the cells were rinsed with 1x Phosphate Buffered Saline (PBS), and fresh CGM were added into the wells. The cells were treated with FITC only for 48 h, and *f*CNP°-CGA in dark at different time points; for 30 min, 6 h, 24 h, and 48 h. The non-treated cells were used as control. The plate was covered with aluminium foil to avoid light exposure. After each time point, the samples were removed from the wells, rinsed with 1× PBS, and 2 mL of fresh CGM was added into each well. The cells were observed under fluorescent microscope, while for flow cytometry analysis, after each time point, the samples were discarded from the wells, rinsed with 1× PBS, and trypsinized. Upon trypsinization, the cells from each well were collected into individual tubes, centrifuged at 1000 rpm for 5 min. The pellets obtained were suspended in 1 mL of MASC buffer (2% FBS, 80% 1× PBS, and EDTA at pH 8) before analyzing in a NovoCyte flow cytometry (ACEA Biosciences, Inc., San Diego, CA, USA).

### 3.13. Statistical Data Analysis

Triplicate independent reads were measured for all the analyses, and expressed as mean (*n* = 3) ± standard deviation (SD). The statistical significant of data was evaluated using Dunnett’s multiple comparison test, One Way Analysis of Variance (ANOVA), GraphPad Prism; at probability value less than 0.05 (*P* < 0.05), with degree of significant denoted with asterisk (*) symbol on.

## 4. Conclusions

Formation of stable, monodispersed, chitosan nanoparticle was achieved through spontaneous ionic gelation between cationic CS and anionic TPP. Nanoparticles obtained were under 100 nm in size, and TPP volume was found to greatly influenced formation of CNP across different formulations; CNP-F1 (0.5 mg/mL CS and 0.7 mg/mL TPP), CNP-F2 (0.25 mg/mL CS and 0.35 mg/mL TPP), and CNP-F3 (0.1 mg/mL CS and 0.2 mg/mL TPP). At lower TPP volumes, inadequate -PO_4_^3−^ were available to form ionic cross-linking with -NH_3_^+^, hence, formation of large sized and unstable particles. Inversely, at higher TPP volumes, stable and monodispersed nanoparticles were formed due to increasing availability of -PO_4_^3−^ to form ionic interactions with CS. The most reproducible, stable and monodispersed CNP° formulation was obtained at optimal parameters consist of 600 µL CS (0.5 mg/mL) at pH 5 and 200 µL TPP (0.7 mg/mL) at pH 2; with an average particle size of 80.89 ± 5.16 nm and a polydispersity index (PDI) of 0.26 ± 0.01.

Encapsulation of the phytochemical CGA into CNP° was initiated through electrostatic interactions between CGA and protonated -NH_3_^+^ of CS. The optimal parameter in synthesizing CGA loaded CNP° was attained at 200 µL of 100 µM CGA (initial concentration), 600 µL of 0.5 mg/mL CS, and 200 µL of 0.7 mg/mL TPP. CNP°-CGA formation using 100 µM CGA led to a prominent increase in CNP° particle size (an increase of 66.20%) with a conserved particle stability and monodispersity (PDI 0.29 ± 0.03) as compared to encapsulation of 10 µM (an increase of 13.72%) and 50 µM (an increase of 2.11%). The significant CGA-L, µM at this optimal parameter was recorded as the highest, 12.04 µM, despite the lowest CGA-EE%, 60.21 ± 0.03% compared to the other two concentrations.

The DPPH scavenging efficacy of CNP°-CGA (IC_50_: 5 µM) was slightly better compared to CGA alone (IC_50_: 6.5 µM), and was able to scavenge the same amount of free radicals at lower CGA concentrations compared to CGA. Apart from that, both CNP° and CGA showed non-toxic properties towards human renal adenocarcinoma, 786-O cells, with percentage of cell viability of 81.37 ± 0.84% and above 100%, respectively. Inversely, CNP°-CGA prompted a better cytotoxic efficacy by reducing cell viability to 60.22% (at 12 µM) after 24 h post-treatment. In addition, the in vitro cellular (human renal adenocarcinoma 786-O cells) accumulation study disclosed that cellular internalization of fluorescently labeled CNP°-CGA begun as early as 30 min and proceeded up to 48 h post-treatment. Inferring, the cationic feature of CNP° shell secured and facilitated the uptake of CGA across the cell membrane.

The synthesized CNP° vector is a good drug delivery system (DDS) candidate for CGA cargo by increasing its in vitro accumulation, thus, improving the bioavailability of CGA and enhancing its antioxidant and cytotoxic properties; hence, potentially lowers the administrative CGA dosages. Furthermore, positive surface charge of CNP° benefited as DDS by accommodating negatively charged drugs, by providing protective shell for the payload, and facilitating the permeability of encapsulated payload across the cellular membrane.

Hitherto, researchers have been widely adapting CNP as a safe option for DDS, and CGA as a therapeutic compound in pharmaceutical industries. CNP° vector plays an importance role in delivering CGA across the membrane, which in turn influences the cellular uptake of CGA, increases its bioavailability, conserves its therapeutic efficacy, and reduces possible occurrence of side effects by reducing the administration dosage of CGA. Nevertheless, the molecular level mechanisms of CGA therapeutic efficacy and CNP as vector, stability of CNP°-CGA, release kinetics of CNP°-CGA, and antioxidant activity, in both in vitro and in vivo studies have yet to be explored further. These in-depth studies are crucial to the design of an efficient chitosan nanoparticle mediated delivery system that could potential protect and enhance the therapeutic efficacy of CGA as a treatment for chronic disease, specifically cancer.

## Figures and Tables

**Table 1:** Chitosan nanoparticles (CNP) synthesis based on three different formulations; CNP-F1, CNP-F2, and CNP-F3. Prior to formation of CNP, CS and TPP solutions were diluted to different concentrations and adjusted to pH 5 and pH 2, respectively. CNP were synthesized through ionic gelation interactions between a fixed volume of CS, 600 µL and different volumes of TPP.

Formulations	Solution	Concentration (mg/mL)	pH	Volume (µL)
CNP-F1	CS	0.50	5	600
	TPP	0.70	2	20, 50, 100, 150, 200, 250, 300
CNP-F2	CS	0.25	5	600
	TPP	0.35	2	20, 50, 100, 150, 200, 250, 300
CNP-F3	CS	0.10	5	600
	TPP	0.20	2	20, 50, 100, 150, 200, 250, 300

**Table 2:** The effects of different CGA final concentrations on polydispersity index, (PDI), average particle size distribution (diameter in nm, nm), and encapsulation efficiency percentage, (EE%) of CNP°. Error bars represent the SD of three independent replicates of the experiment.

Final Concentration of CGA in CNP	Average Particle Size Distribution, (nm)	Polydispersity Index, (PDI)	Encapsulation Efficiency, (EE %)	CGA Loading, (CGA-L, µM)
0 µM (CNP°)	80.89 ± 5.16	0.26 ± 0.01	-	-
2 µM	91.99 ± 18.28	0.30 ± 0.04	74.43 ± 0.31	1.49
10 µM	82.60 ± 15.81	0.28 ± 0.06	62.30 ± 0.05	6.23
20 µM	134.44 ± 18.29	0.29 ± 0.03	60.21 ± 0.03	12.04

**Table 3:** Summary of functional groups present in FTIR spectra of CS, TPP, CNP°, CGA, and CNP°-GCA.

Functional Group	Samples	Wavenumber (cm^−1^)	Transmittance Percentage (%)	Figure 9 Label
**-OH/-NH_2_ stretching vibration**	CS	3353.91	35.26	A
	TPP	3253.92	57.22	B
	CNP°	3364.97	34.74	C
	CNP°-CGA	3398.61	44.97	D
**N-H bending, amide**	CS	1587.00	52.24	E
	CNP°	1527.82	72.08	F
	CNP°-CGA	1520.64	70.60	G
**Stretching of C=O, amide**	CS	1641.06	51.11	H
	CNP°	1630.94	52.27	I
**Stretching of P=O**	TPP	1126.89	20.92	J
	CNP°	1073.91	32.03	K
	CNP°-CGA	1014.92	15.61	L
**Asymmetrical stretching vibration of P-O-P**	TPP	883.70	22.73	M
	CNP°	948.77	49.86	N
	CNP°-CGA	949.14	41.68	O
**C=O stretching vibration of carboxyl and ester group**	CGA	1687.62	17.02	P
	CNP°-CGA	1700.27	16.99	Q
**Aromatic ring C=C stretching vibration**	CGA	1439.88	49.94	R
	CNP°-CGA	1428.52	52.90	S
**Stretching vibration of C-O-C and C-O of carboxyl and ester group**	CGA	1279.45, 1185.17	13.86, 10.39	T
	CNP°-CGA	1265.04, 1228.71	30.34, 30.00	U
**Bending of C-H and COH**	CGA	1119.50	12.37	V
	CNP°-CGA	1163.43	31.83	W
**CH aromatic bending**	CGA	810.46	53.71	X
	CNP°-CGA	812.13	70.81	Y

## Supplementary Materials

Supplementary materials can be found at https://www.mdpi.com/1422-0067/20/19/4667/s1.

## Abbreviations

µL	Microliter
μM	Micromolar
5-CQA	5-O-caffeoylquinic acid
786-O	Human renal cancer cell line
ANOVA	One Way Analysis of Variance
ARE	Antioxidant response element
°C	Degree Celsius
CGA	Chlorogenic acid
CGA-EE%	CGA encapsulation efficacy
CGA-L	CGA loading
CGM	Complete growth media
cm	Centimeter
CNP	Chitosan nanoparticles
CNP°	Chitosan nanoparticles at optimum parameter
CNP°-CGA	Chitosan nanoparticles-encapsulated chlorogenic acid at optimal parameter
CNP-F1	Chitosan nanoparticle-Formulation 1
CNP-F2	Chitosan nanoparticle-Formulation 2
CNP-F3	Chitosan nanoparticle-Formulation 3
CO2	Carbon dioxide
CS	Chitosan
DDS	Drug delivery systems
DLS	Dynamic light scattering
DMSO	Dimethyl sulfoxide
DNA	Deoxyribonucleic acid
DPPH	1,1-Diphenyl-2-picrylhydrazine
EDTA	Ethylenediaminetetraacetic acid
EE%	Encapsulation efficiency percentage
EPR	Enhanced permeability and retention effect
FBS	Fetal Bovine Serum solution
fCNP°	FITC labeled chitosan nanoparticles
fCNP°-CGA	FITC labeled chitosan nanoparticles-encapsulated chlorogenic acid
FESEM	Field emission scanning electron microscope
FITC	Fluorescein 5(6)-isothiocyanate
FTIR	Fourier-transform infrared spectroscopy
GST	Glutathione S-transferases
HCl	Hydrochloric acid
HeLa	Human epithelial cell line
IC50	Concentration of a sample required to reduce the biological target by 50%
MCF-7	Breast cancer cell line
MDA-MB-435	Breast cancer cell line
Mg	Milligram
mL	Milliliter
mM	Millimolar
MTT	3-(4, 5-dimethylthiazol-2-yl)-2, 5-diphenyltetrazolium bromide
NaCO3	Sodium carbonate
NaOH	Sodium hydroxide
NF-κB	Nuclear factor κB
NH2	Primary amine functional groups
-NH3+	Protonated primary amine groups of CS
NIH 3T3	Mouse fibroblast NIH 3T3 cell line
NQO1	NAD(P)H:quinone oxidoreductase 1
nm	Nanometer
OH	Hydroxyl
OH-	Hydroxide ions
PBS	Phosphate buffered saline
PDI	Polydispersity index
pH	potential of Hydrogen
-PO43-	Anionic phosphate groups
RNA	Ribonucleic acid
ROS	Reactive oxygen species
rpm	Revolutions per minute
RPMI	Roswell Park Memorial Institute
SD	Standard deviation
sdH2O	Sterile distilled water
SDS	Sodium dodecyl sulfate
siRNA	Small interfering RNA
TEM	Transmission electron microscopy
TNBS	2,4,6-trinitrobenzene sulfonic acid
TPP	Sodium tripolyphosphate
UV	Ultraviolet
