# Supplementary material for: Increased ROS Scavenging and Antioxidant Efficiency of Chlorogenic Acid Compound Delivered via a Chitosan Nanoparticulate System for Efficient In Vitro Visualization and Accumulation in Human Renal Adenocarcinoma Cells
Source: Int J Mol Sci. 2019 Sep 20;20(19):4667. doi: 10.3390/ijms20194667 (PMC6801874; doi:10.3390/ijms20194667)
Supplement: Supplementary file 1 [file ijms-20-04667-s001.pdf]

Untreated (A)

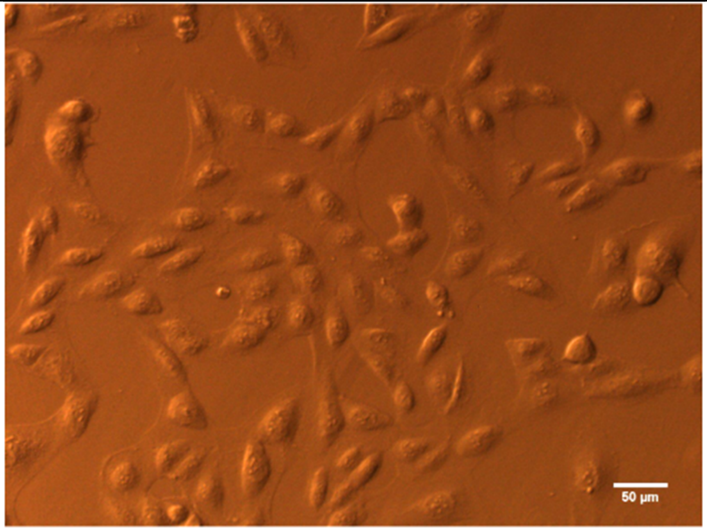

Brightfield (1)

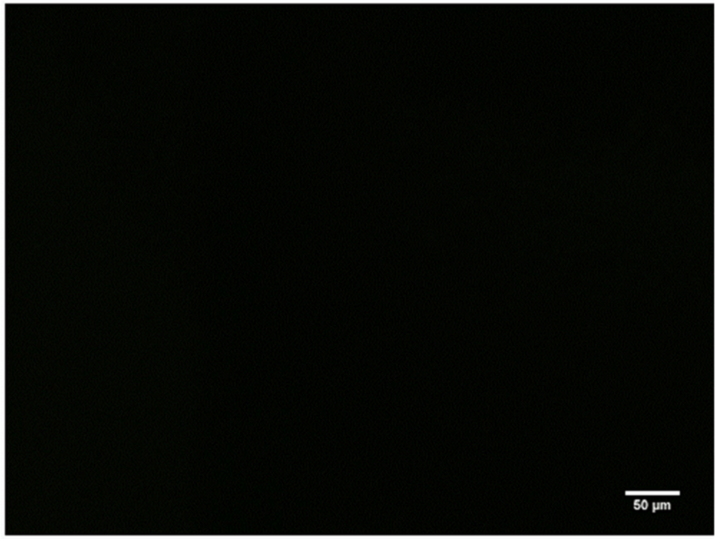

FITC (2)

c

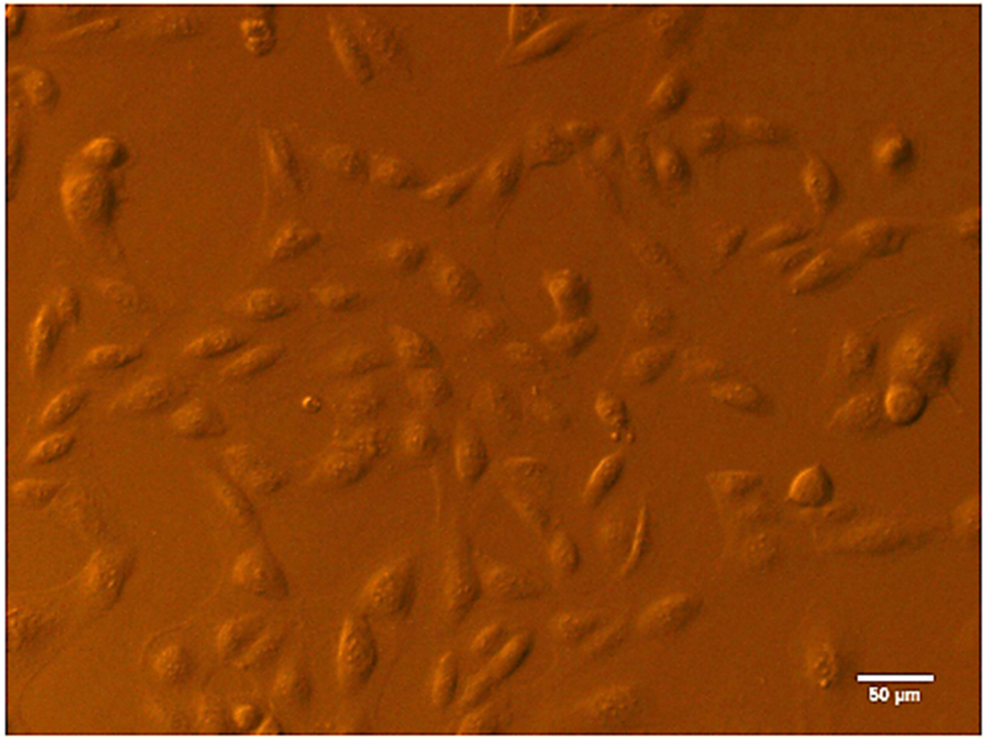

Merged (3)

Scale: 50 µm

FITC only (B)

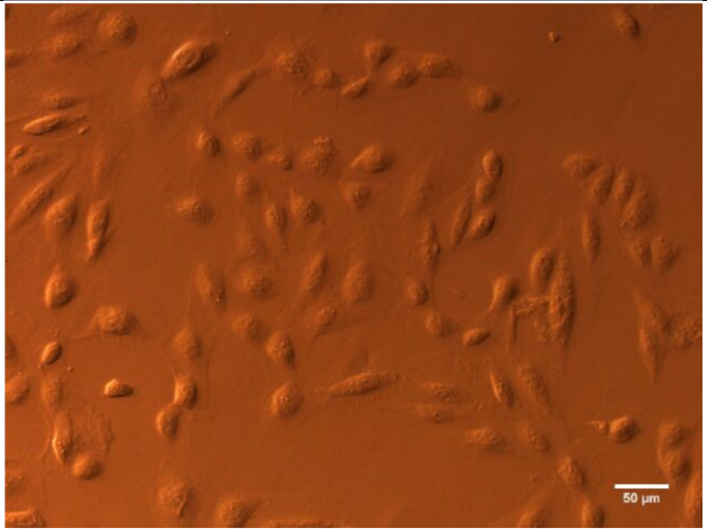

Brightfield (1)

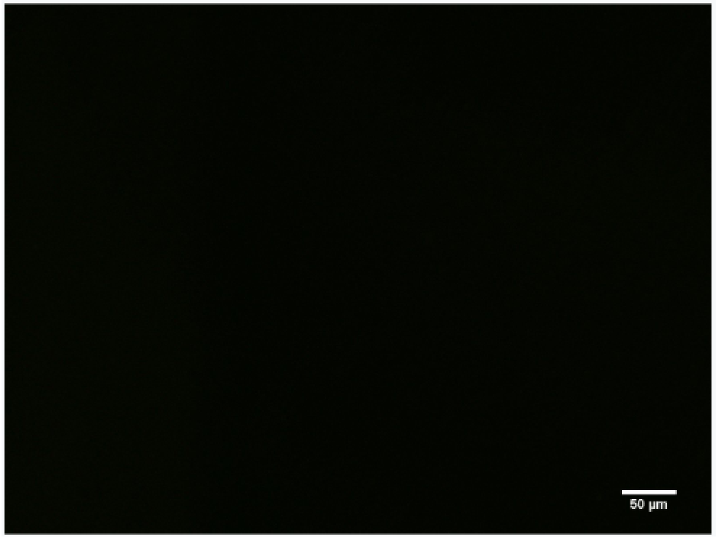

FITC (2)

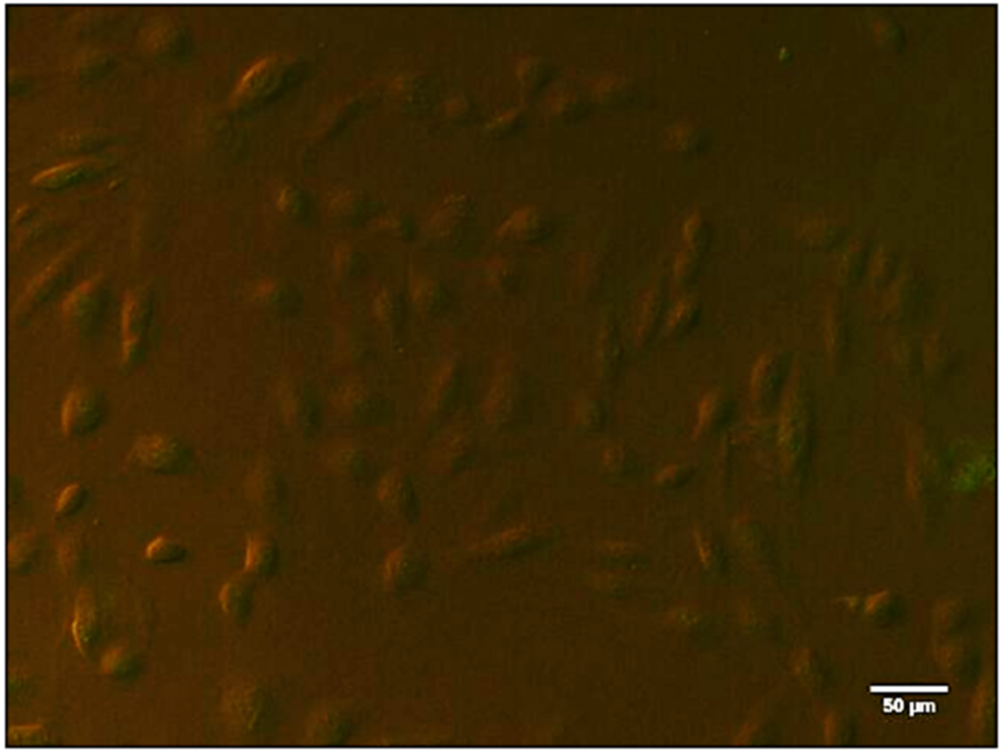

Merged (3)

Scale: 50 µm

30 minutes (C)

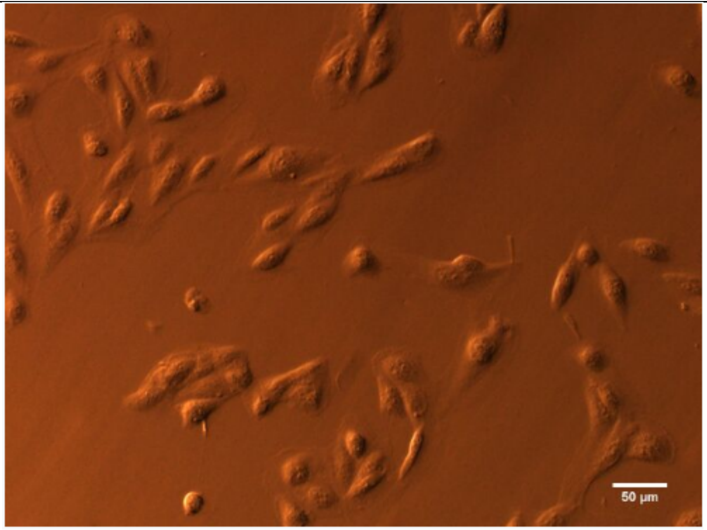

Brightfield (1)

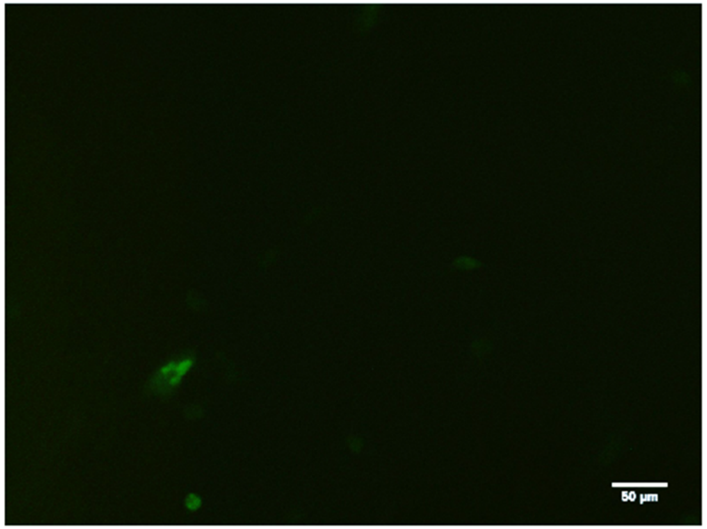

FITC (2)

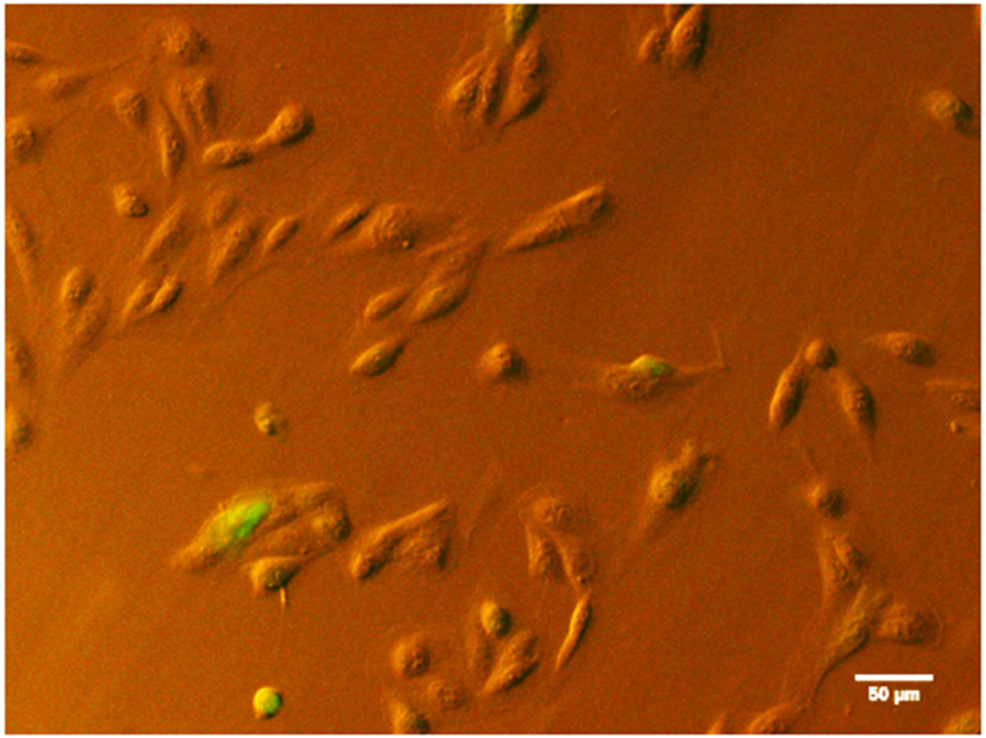

Merged (3)

Scale: 50 µm

6 hours (D)

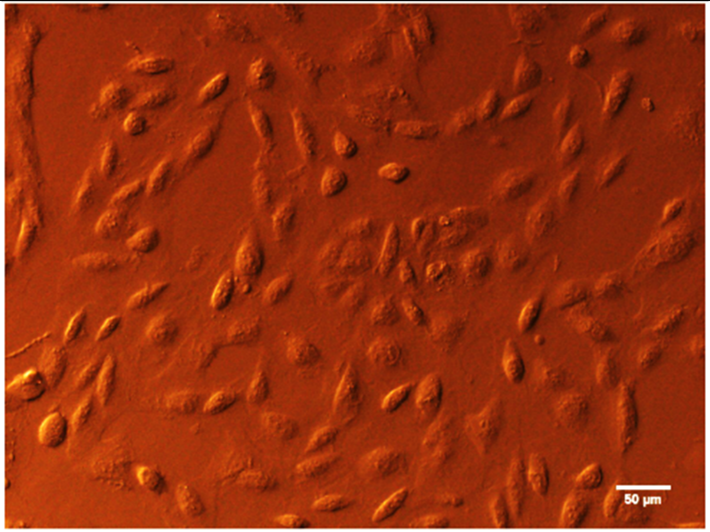

Brightfield (1)

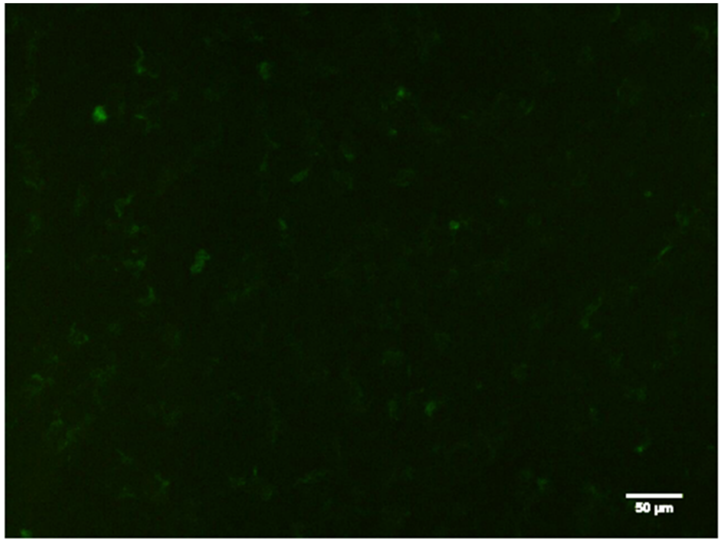

FITC (2)

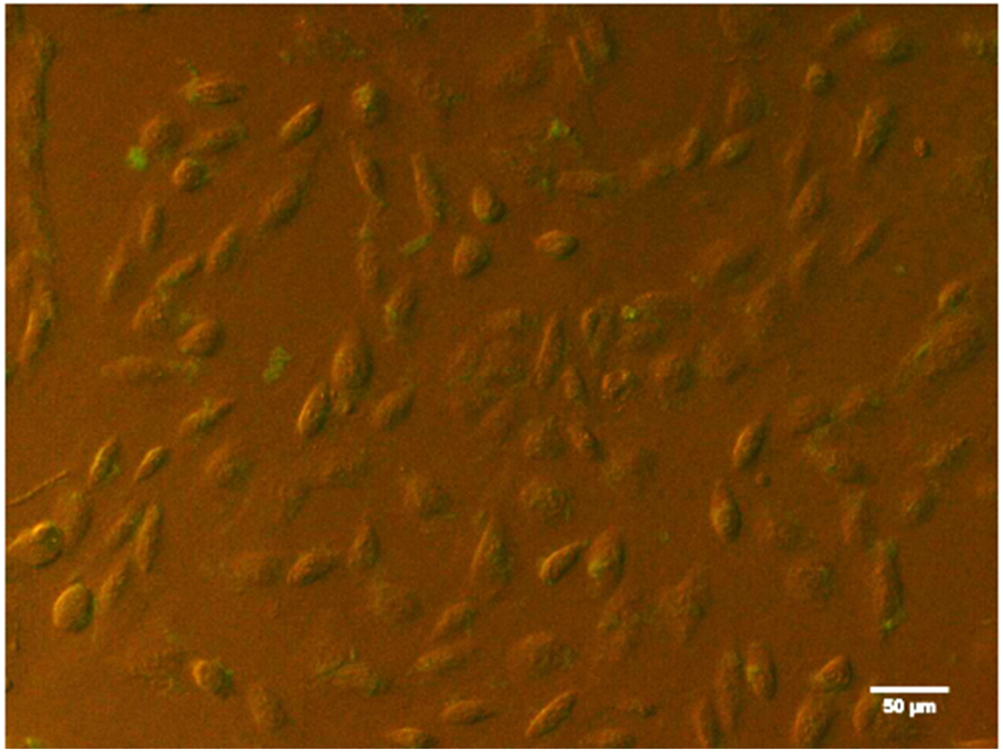

Merged (3)

Scale: 50 µm

24 hours (E)

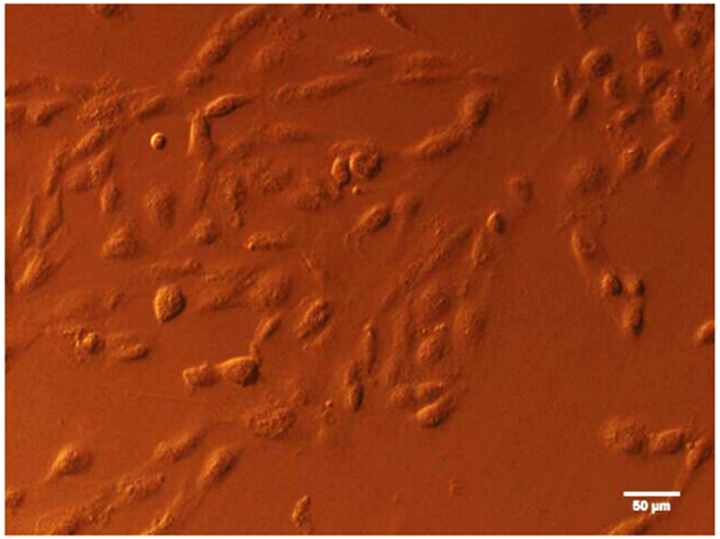

Brightfield (1)

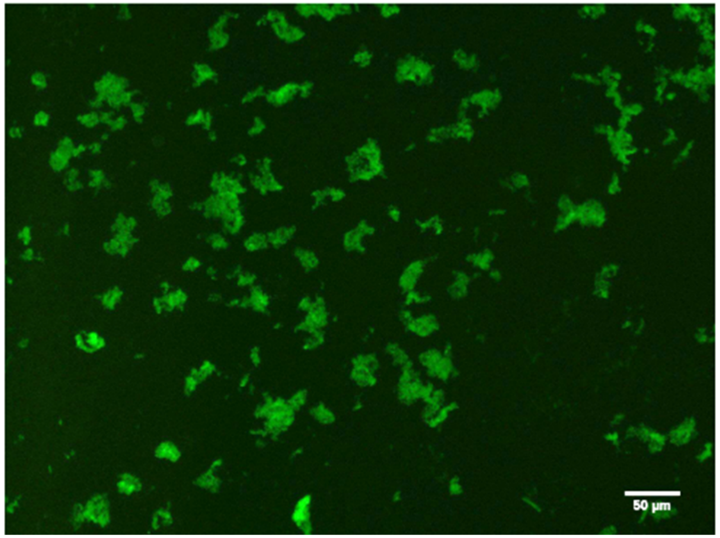

FITC (2)

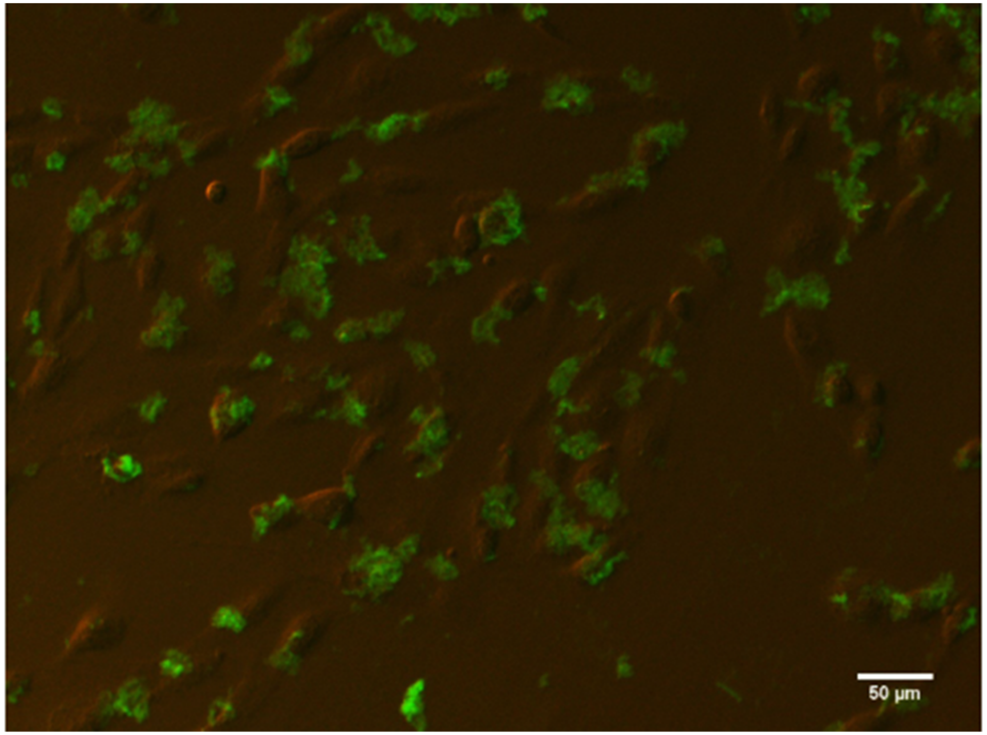

Merged (3)

Scale: 50 µm

48 hours (F)

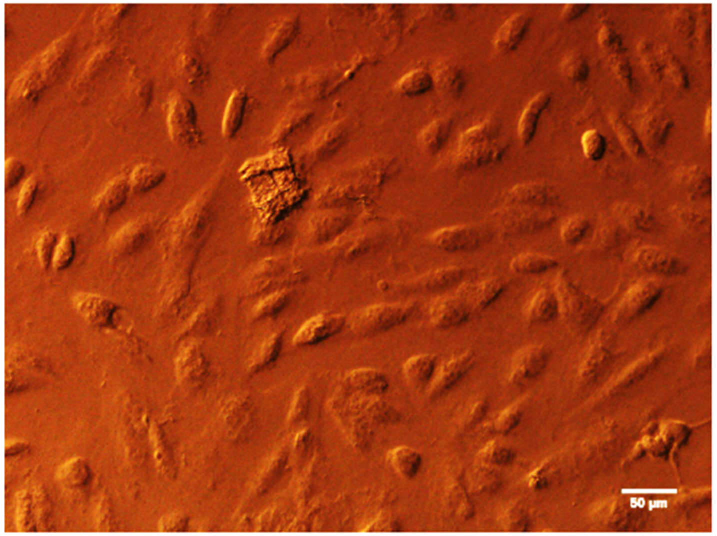

Brightfield (1)

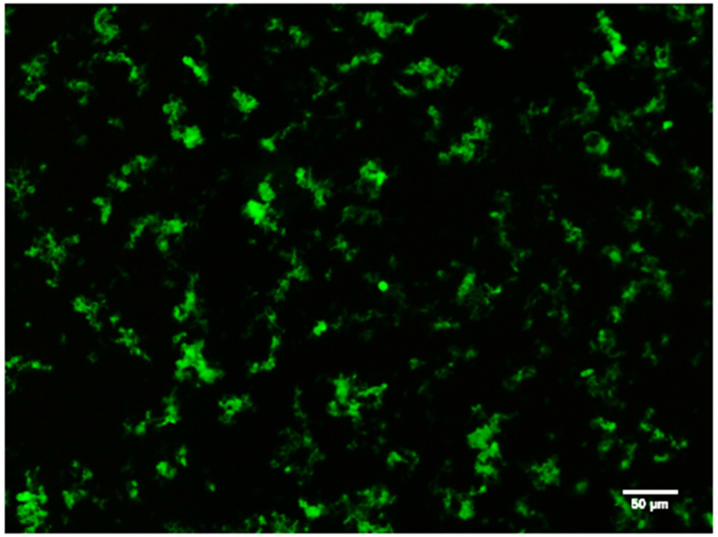

FITC (2)

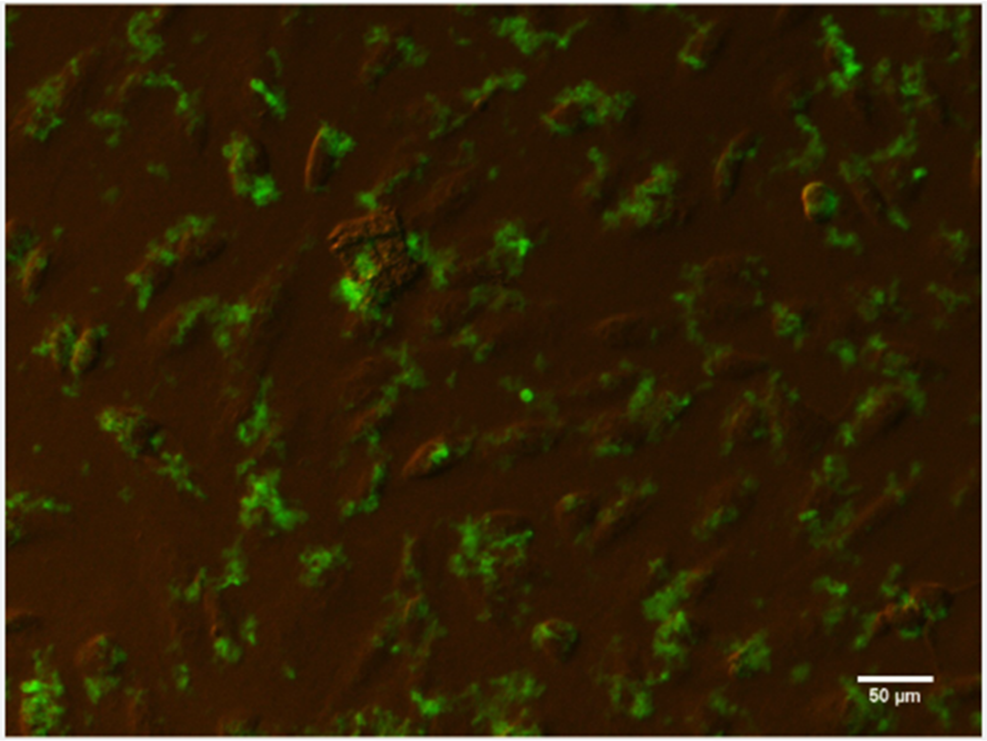

Merged (3)

Scale: 50 µm

Supplementary 1. Fluorescent microscopic study of in vitro accumulation of fCNP°-CGA in 786-O0 cells at 30 minutes, 6, 24, and 48 hours. Merged image (column 3) represents the merged images of brightfield image (column 1) and FITC image (column 2). The accumulation of fCNP°-CGA in 786-O cells showed a time-dependent trend, indicated through the increase of fluorescent intensity over time. Accumulation of fCNP°-CGA was observed as early as 30 minutes.
